# NKX2-5 regulates vessel remodeling in scleroderma-associated pulmonary arterial hypertension

**DOI:** 10.1172/jci.insight.164191

**Published:** 2024-04-23

**Authors:** Ioannis Papaioannou, Athina Dritsoula, Ping Kang, Reshma S. Baliga, Sarah L. Trinder, Emma Cook, Xu Shiwen, Adrian J. Hobbs, Christopher P. Denton, David J. Abraham, Markella Ponticos

**Affiliations:** 1 Division of Medicine, Department of Inflammation, University College London, Royal Free Campus, London, United Kingdom.; 2 William Harvey Research Institute, Barts and The London School of Medicine & Dentistry, Queen Mary University of London, Charterhouse Square, London, United Kingdom.

**Keywords:** Pulmonology, Vascular biology, Cardiovascular disease, Hypertension

## Abstract

NKX2-5 is a member of the homeobox-containing transcription factors critical in regulating tissue differentiation in development. Here, we report a role for NKX2-5 in vascular smooth muscle cell phenotypic modulation in vitro and in vascular remodeling in vivo. NKX2-5 is upregulated in scleroderma patients with pulmonary arterial hypertension. Suppression of NKX2-5 expression in smooth muscle cells halted vascular smooth muscle proliferation and migration, enhanced contractility, and blocked the expression of extracellular matrix genes. Conversely, overexpression of NKX2-5 suppressed the expression of contractile genes (*ACTA2*, *TAGLN*, *CNN1*) and enhanced the expression of matrix genes (*COL1*) in vascular smooth muscle cells. In vivo, conditional deletion of NKX2-5 attenuated blood vessel remodeling and halted the progression to hypertension in a mouse chronic hypoxia model. This study revealed that signals related to injury such as serum and low confluence, which induce NKX2-5 expression in cultured cells, is potentiated by TGF-β and further enhanced by hypoxia. The effect of TGF-β was sensitive to ERK5 and PI3K inhibition. Our data suggest a pivotal role for NKX2-5 in the phenotypic modulation of smooth muscle cells during pathological vascular remodeling and provide proof of concept for therapeutic targeting of NKX2-5 in vasculopathies.

## Abstract 2

NKX2-5 is a key determinant of phenotypic modulation in smooth muscle cells, and NKX2-5 deficiency ameliorates experimental hypertension and vascular remodeling in a hypoxia-driven mouse model.

## Graphical abstract

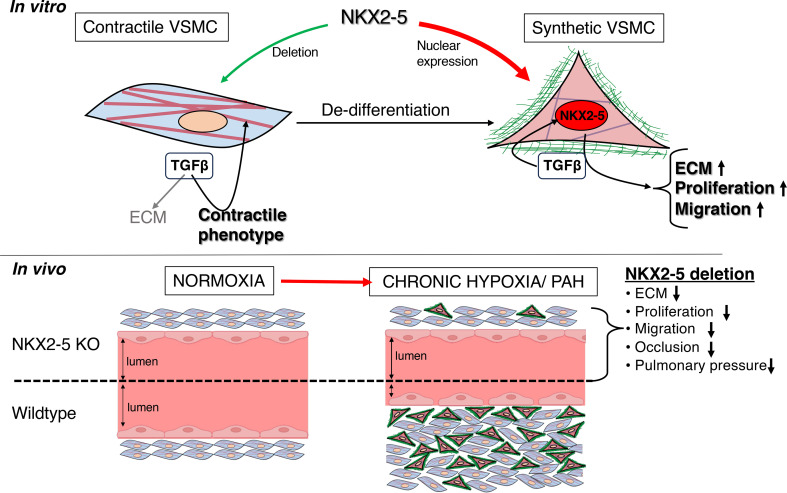

## Introduction

Vascular remodeling can be defined as the adaptive structural rearrangement of blood vessels through changes in cell growth, survival, and migration, and modulation of the extracellular matrix (ECM) (1–4). Vascular remodeling is involved in embryonic angiogenesis and arteriogenesis. In mature vessels, however, it is a consequence of vascular injury and diseases such as atherosclerosis and pulmonary arterial hypertension (PAH). In adult healthy blood vessels, vascular smooth muscle cells (VSMCs) have a low rate of proliferation and ECM synthesis, are largely nonmigratory, and are committed to carrying out a contractile function.

However, upon vascular injury, contractile VSMCs temporarily undergo phenotypic modulation to a highly proliferative synthetic phenotype, which is vital in replacing lost VSMCs during vascular repair. The same phenotypic modulation, however, can also contribute to vascular pathology (5) by altering vascular structure in a way that impedes function. This pathologic vascular remodeling is a critical component of most vascular diseases. Heretofore, we shall refer to this proliferative, biosynthetic phenotype as the “synthetic” phenotype.

Vascular remodeling is of importance in PAH. PAH, defined as a resting pulmonary artery blood pressure of 25 mmHg or greater, results in dramatic arterial structure changes, most notably endothelial dysfunction and overproliferation of the smooth muscle layer, leading to vessel remodeling and narrowing of the vessel lumen (6–8). As this remodeling process continues, it causes a progressive increase in pulmonary vascular resistance, right ventricular (RV) hypertrophy, and ultimately right heart failure. The marked remodeling of the pulmonary arteries is believed to result from matrix deposition and proliferation of existing smooth muscle cells. Matrix deposition and proliferation are the hallmarks of the synthetic VSMC phenotype. Chronic hypoxia–induced PAH has been modeled using mice and reproducibly demonstrates many of the features that occur in human PAH.

The molecular mechanism regulating VSMC phenotypic modulation is incompletely understood. Reports have implicated a number of key transcription factors such as SRF (switching from contractile to the synthetic phenotype) and myocardin (migration and proliferation), KLF4 (dedifferentiation to the synthetic phenotype), and ZEB1 (collagen synthesis repressor maintaining the contractile phenotype) (9–12), yet many questions remain. In vitro, phenotypic switch of cultured VSMCs to the contractile phenotype can be effectively induced by serum deprivation or high confluence, which over a period of several days results in strongly increased expression of contractile machinery components and a concomitant reduction in ECM synthesis (5, 13).

More recently, seminal work by Chen et al. (13) uncovered a role for FGF2 and TGF-β in the maintenance of the contractile phenotype. It appears that TGF-β signaling is essential for the expression of contractile markers and FGF2 exerts direct control over the pathway. Blocking TGF-β prevents cells cultured in serum from reactivating their contractile markers upon serum removal.

NKX2-5 belongs to the highly conserved NK-2 family of homeobox DNA-binding transcription factors (14). Expression of NKX2-5 is one of the earliest markers of the cardiac lineage and is first observed in the developing embryonic heart (15). Targeted disruption of NKX2-5 in mice is lethal, causing the arrest of heart development after the initial stage of cardiac looping (16). A critical role for NKX2-5 in blood vessel development has also been discovered; NKX2-5 is essential for the formation of the major pharyngeal vessels (17). After development, NKX2-5 expression is heart-limited, with undetectable expression in most other tissues such as the lung.

The *COL1A2* gene (encodes the pro-α2 chain of type I collagen) is the rate-limiting component for the synthesis of collagen I (COL1). Its expression is regulated by 2 key elements, the promoter and an enhancer (18), which respectively control low-level expression for maintenance and high-level expression required for ECM repair or remodeling. Many different cell types, particularly proliferating cells, produce COL1 for maintenance and hence the promoter is active in a large variety of tissues. The enhancer, on the other hand, is only activated in a handful of mesenchymal cells, such as activated fibroblasts that need to produce large amounts of ECM (18).

Previously, we discovered, that NKX2-5 expression can be detected in adult collagen-producing VSMCs in vitro and that it is, in fact, required for activating the *COL1A2* enhancer and driving high-level collagen expression in VSMCs (18). Given that VSMCs only express high levels of collagen type Iα2 when they swap to a synthetic phenotype, we investigated the relevance of NKX2-5 reactivation in adult VSMCs with respect to VSMC phenotypic modulation. We report that NKX2-5 can indeed be readily detected specifically in synthetic VSMCs and not contractile VSMCs, coinciding with high levels of collagen type Iα2 expression. We also report that NKX2-5 can be detected in adult remodeled blood vessels from PAH patients, but not in healthy blood vessels, while conditional NKX2-5 deletion inhibits adaptive vascular remodeling in a mouse model of pulmonary hypertension. We propose that PI3K/AKT and ERK5 signaling in response to injury or disease, potentiated by TGF-β signaling, reactivates NKX2-5 in adult blood vessels, enabling some of the cells within the intima to switch to the synthetic phenotype. Although by necessity most of the cells need to remain contractile to maintain vascular tone, having some of the VSMCs become synthetic facilitates proliferation and repair. Yet, in doing so, NKX2-5 also licences the overgrowth of the smooth muscle layer, allowing chronic damage signals to induce vascular remodeling. This proposal is supported by our recent finding that NKX2-5 promoter polymorphisms are genetically associated with pulmonary hypertension in scleroderma patients (19).

## Results

### NKX2-5 expression in human vascular remodeling.

**Figure 1:**
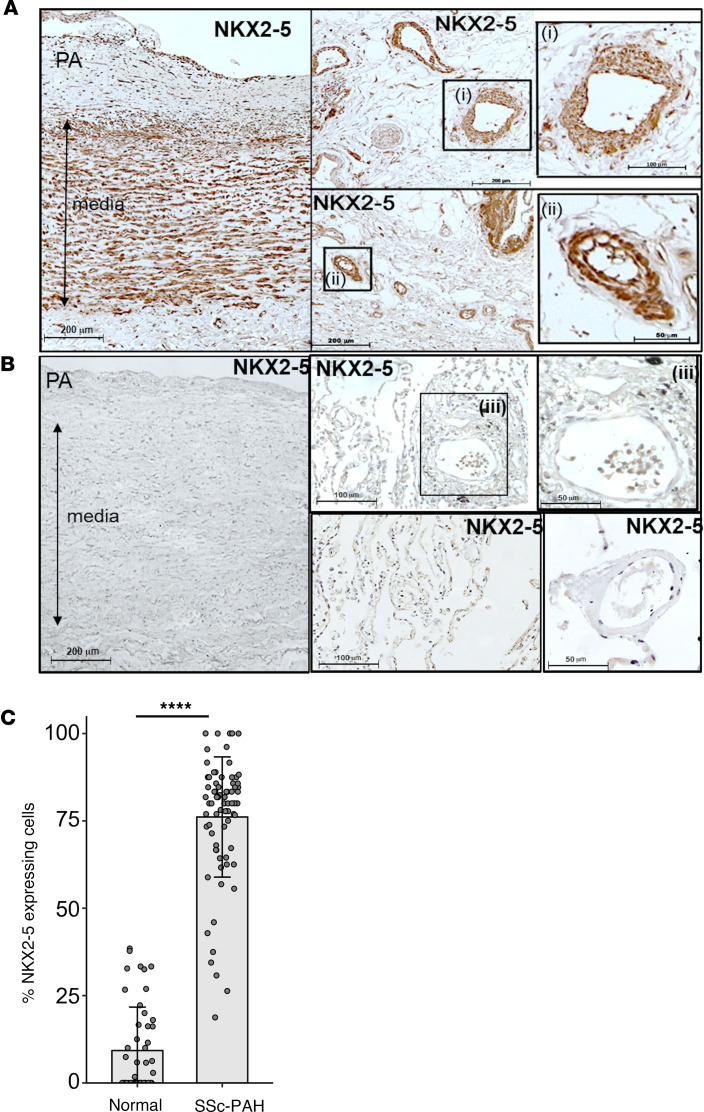
NKX2-5 is expressed by VSMCs in scleroderma patients with PAH (SSc-PAH). Lung tissue sections were immunostained for NKX2-5 (brown) and counterstained with hematoxylin. Examples of large pulmonary arteries (PA) (>100 μm, left panel and inset i) and medium (40–70 μm, inset ii) are highlighted, but small arteries (20–40 μm) can also be seen. (**A**) SSc-PAH patients. (**B**) Healthy lung tissue. (**C**) NKX2-5 expression was quantified by counting positive cells in sections of PAH or control pulmonary tissue and expressed as a percentage of total cells. For each condition, 75 vessels 20–100 μm in diameter were counted in total from 5 different patients. ****P < 0.0001 by paired, 2-tailed Student’s *t* test. Data presented as mean ± SD.

We have identified NKX2-5 as an important regulator of collagen 1α2 expression in VSMCs (18) through direct activation of the *COL1A2* far upstream enhancer, a crucial regulatory element of COL1 known to be associated with fibrotic disease (20–22). Postmortem tissue from patients with PAH was used to test the hypothesis that NKX2-5 is expressed in diseased vessels or vessels undergoing vascular remodeling (Figure 1). Using validated specific antibodies (Supplemental Figure 1; supplemental material available online with this article; https://doi.org/10.1172/jci.insight.164191DS1), we observed NKX2-5 expression in the pulmonary vasculature of patients with hypertension (Figure 1) as well as in diseased carotid, coronary, and peripheral arteries (Supplemental Figure 2), where it coincides with ACTA2 expression in VSMCs (Supplemental Figure 2, F and G). In patients with PAH, NKX2-5 was expressed in the media and neointima of large pulmonary arteries (Figure 1A), medium-sized muscularized arterioles (Figure 1A), and small muscularized arterioles (Figure 1A). No expression was observed in normal vessels (Figure 1, B and C). Quantification of NKX2-5 expression revealed that over 70% of cells expressed NKX2-5 in PAH patient pulmonary arteries/arterioles (20–100 μm in diameter) compared with less than 10% of cells in vessels from control lungs (Figure 1C).

### NKX2-5 mediates phenotypic modulation of VSMCs.

Transition of the normal contractile VSMCs in the arterial media toward the synthetic phenotype is associated with gradual upregulation of several ECM genes such as *COL1*, connective tissue growth factor (*CCN2*), and fibronectin (*FN1*). Concomitantly, the expression of proteins that are components of the contractile machinery such as ACTA2, TAGLN, CNN1, smooth muscle myosin heavy chain (MYH11), and smoothelin (SMTN) are gradually downregulated.

**Figure 2:**
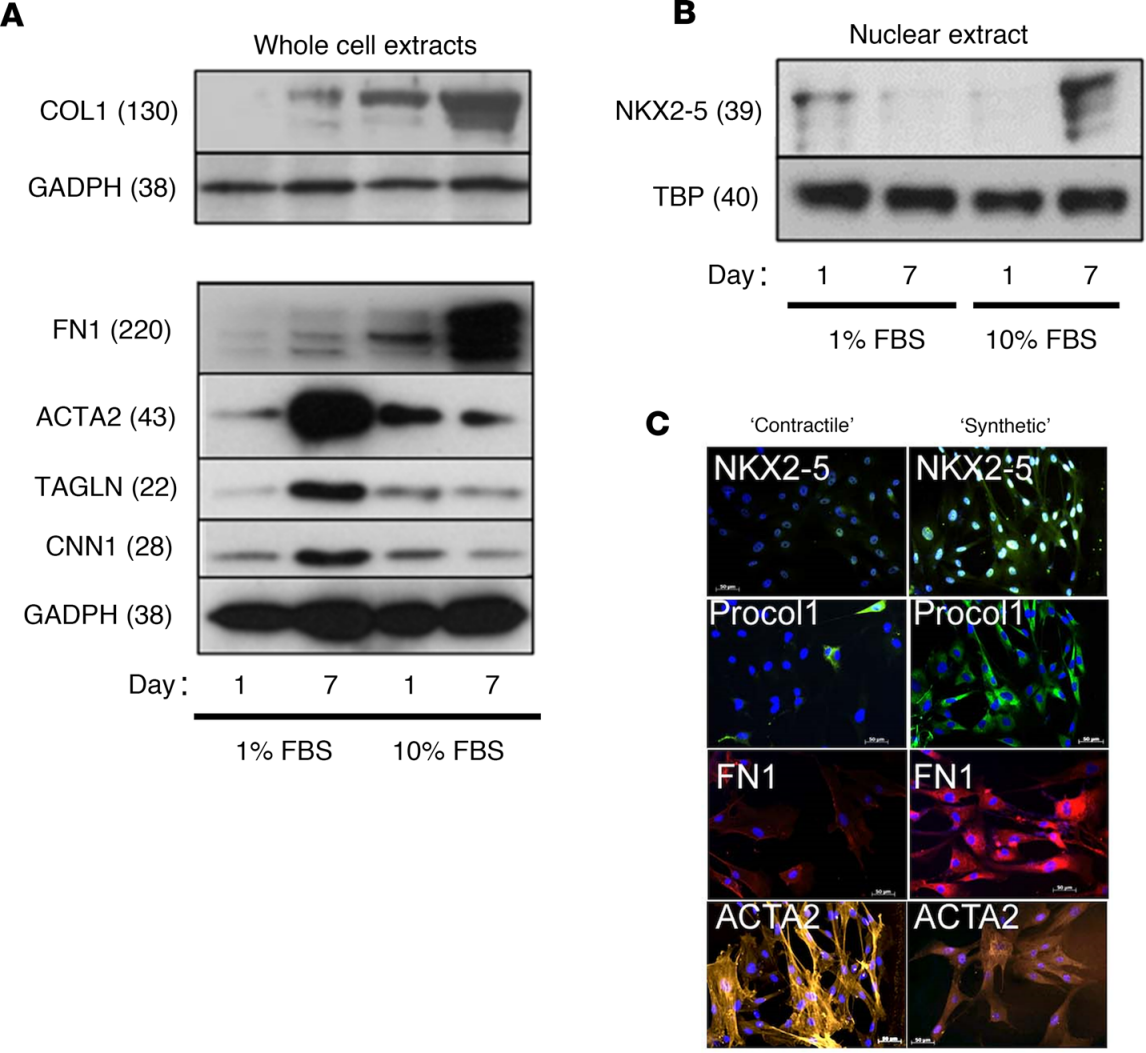
NKX2-5 expression is associated with the synthetic phenotype in HPASMCs. HPASMCs were cultured in vitro under conditions favoring the contractile or synthetic phenotype over 7 days. (**A**) Protein expression levels of NKX2-5, synthetic cell markers COL1, and FN1, and contractile cell markers ACTA2, CNN1, and TAGLN were determined by Western blotting. A representative blot from 3 independent experiments is shown. Statistical significance was examined by 2-way ANOVA (Supplemental Figure 8). The effect of FBS and the effect of time were both significant at *P* < 0.05 for all genes. All genes come from the same samples run on different, but concurrent, blots, except for COL1, which was run on a separate occasion. (**B**) Nuclear extracts were used to quantify NKX2-5 expression via Western blotting with the E1Y8H antibody. A representative blot from 2 independent experiments is shown. Substantial expression of NKX2-5 was only observed after prolonged culture in serum (*P* < 0.05 via 2-tailed Student’s *t* test). NKX2-5 and TBP were run on different, but concurrent, blots. (**C**) Immunofluorescence was carried out on contractile (7 days in 1% FBS) or synthetic (7 days in 10% FBS) HPASMCs using specific antibodies for NKX2-5 (Alexa Fluor 488, green), intracellular pro-collagen type I (Procol1, Alexa Fluor 488, green), FN1 (Alexa Fluor 594, red), ACTA2 (Cy3, orange), and DAPI (blue). Nuclear expression of NKX2-5 was concomitant with high COL1 and FN1 expression in synthetic, but not contractile, HPASMCs. High levels of organized ACTA2 expression were only visible in contractile cells. Scale bars: 50 μm. All protein molecular weights are given as kDa in parentheses.

We cultured human VSMCs in vitro under conditions favoring either the contractile or synthetic phenotype. Data are shown only for human pulmonary arterial smooth muscle cells (HPASMCs) (Figure 2), but human aortic smooth muscle cells behaved in a very similar manner (data not shown). Western blot analyses of total and nuclear lysates revealed that contractile protein (ACTA2, TAGLN, CNN1) downregulation and ECM protein (COL1, FN1) upregulation paralleled the expression of nuclear NKX2-5 (Figure 2, A and B). These findings were confirmed by immunofluorescence. Synthetic cells expressed nuclear NKX2-5, high levels of intracellular pro-collagen type I (Procol1) and FN1, but low levels of ACTA2 (Figure 2C). Conversely, contractile cells expressed little NKX2-5, Procol1, and FN1, but high levels of ACTA2, organized into distinct cytoplasmic myofilaments (Figure 2C). Culturing the cells under conditions promoting the synthetic phenotype resulted in substantial morphological changes. While cells with low expression of NKX2-5 remained mostly unchanged, those with higher nuclear NKX2-5 levels also stained strongly for Procol1 and the cells become larger with obvious nucleoli, suggesting increased levels of euchromatin (Supplemental Figure 3). A shape change also became evident as NKX2-5 accumulated. Initially, the cells were flatter and more spread out, suggesting increased matrix interaction, but gradually as nuclear NKX2-5 accumulated they became thinner and spindle shaped, with multiple pseudopodia, an indication of increased motility (Supplemental Figure 4).

**Figure 3:**
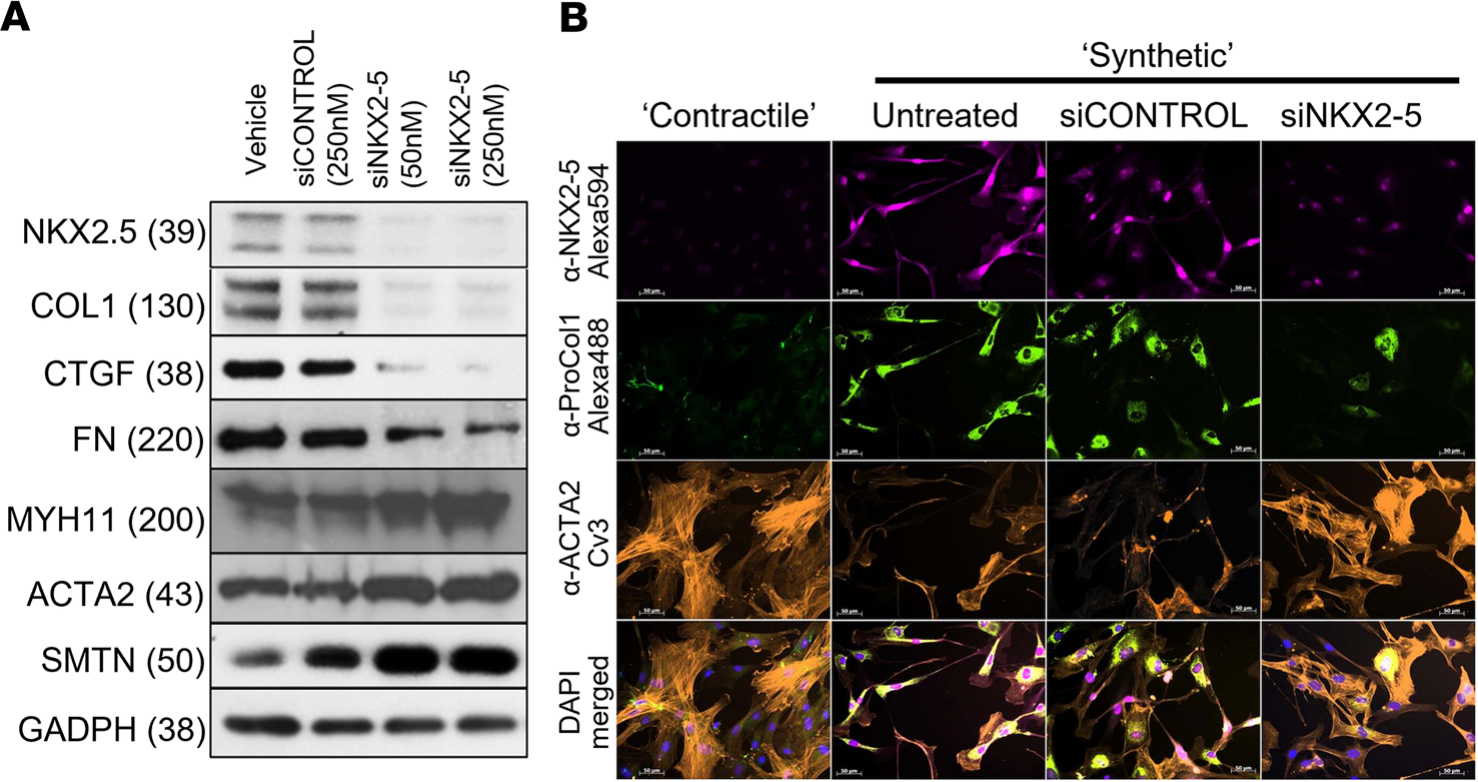
NKX2-5 expression is needed to maintain the contractile phenotype. HPASMCs cultured in serum for 7 days were treated with siRNA against NKX2-5 (50 nM or 250 nM) or scrambled siCONTROL (250 nM) for 72 hours. The densitometry results were analyzed by 1-way ANOVA with Tukey’s post hoc test (Supplemental Figure 9). The effect of siNKX2-5 was significant at *P* < 0.05 for all genes. (**A**) Representative Western blot (*n* = 3) for contractile and synthetic markers (NKX2-5 antibody: E1Y8H). NKX2-5 downregulation suppresses COL1, CTGF, and FN1, but increases MYH11, ACTA2, and SMTN. For all proteins, the same samples were run on different, but concurrent, Western blots. (**B**) The expression of NKX2-5, Procol1, and ACTA2 after NKX2-5 knockdown was investigated by immunofluorescence. NKX2-5 knockdown resulted in reduced Procol1 expression, while ACTA2 expression and organization into myofilaments was partially restored. Scale bars: 50 μm. All protein molecular weights are given as kDa in parentheses.

We further probed the role of NKX2-5 in promoting these changes in gene expression and function using RNA interference (siRNA) (Figure 3A). Cells grown under conditions that favor the synthetic phenotype and expressing high levels of NKX2-5 were treated with either nontargeting siRNA (siCONTROL, 250 nmol/L) or NKX2-5–specific siRNA (siNKX2-5, 50 nmol/L or 250 nmol/L). Knockdown of NKX2-5 expression downregulated synthetic markers (COL1, CCN2, FN1) and upregulated contractile markers (MYH11, ACTA2, SMNT).

NKX2-5 knockdown altered contractile and synthetic VSMC morphology (Figure 3B). Untreated VSMCs and those treated with siCONTROL expressed high levels of nuclear NKX2-5, high levels of Procol1, and lower levels of ACTA2, with disorganized cytoplasmic actin fibrils and pseudopodial protrusions, when cultured under synthetic conditions. However, knockdown of NKX2-5 (siNKX2-5) rescued the contractile phenotype, decreasing the levels of Procol1 while increasing ACTA2 levels and organization.

### Constitutive overexpression of NKX2-5 inhibits the upregulation of contractile markers and upregulates collagen.

**Figure 4:**
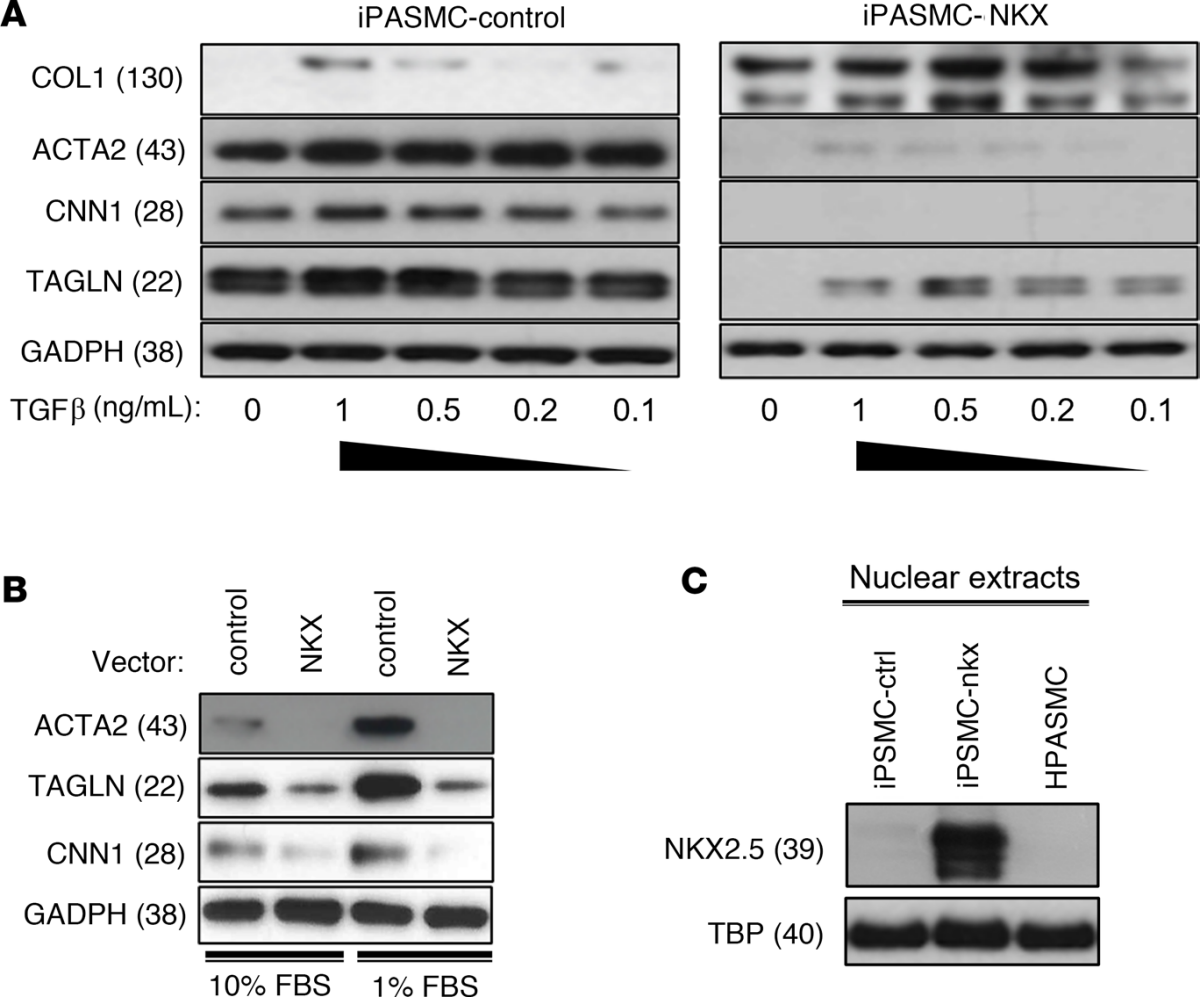
NKX2-5 expression locks SMCs into the synthetic phenotype. (**A**) An immortalized HPASMC (iPASMC) cell line was transduced with an NKX2-5 lentiviral vector (iPASMC-NKX) or a control vector (iPASMC-control). The transduced cells were cultured for 7 days in growth media with 1% FBS and various concentrations of TGF-β to induce contractile marker expression. Evaluation of these results by 2-way ANOVA (Supplemental Figure 10) confirmed that the differences between control and transduced cells were highly significant (*P* < 0.01). Control and NKX2-5 samples were always run on the same blot. For all proteins, the same samples were run on different, but concurrent, Western blots. (**B**) Primary HPASMCs were transduced with either an NKX2-5 lentiviral vector (NKX) or a control vector. After transduction and selection, the cells were cultured for 7 days in media either at 10% FBS and low density to induce the synthetic phenotype or 1% FBS and high density to activate the contractile phenotype. Two-tailed Student’s *t* test analysis (*n* = 2) showed reduction in ACTA2 and TAGLN protein expression after NKX2-5 transduction was significant in both 1% and 10% FBS (*P* < 0.01), but the CNN1 reduction was only significant in 1% FBS (*P* < 0.01). (**C**) Western blot analysis of NKX2-5 expression in nuclear extracts from iPASMC-NKX and iPASMC-control (iPASMC-ctrl). Contractile HPASMCs are also included as a reference. In **B** and **C**, different genes were analyzed in the same samples run on different, but concurrent, blots.

Further evidence supporting a causative relationship between NKX2-5 and the contractile phenotype was obtained by stably overexpressing NKX2-5 in an immortalized HPASMC cell line (iPASMC) (Figure 4A). The expression of NKX2-5 in this system is much higher than physiological levels, to make the repression and suppression of NKX2-5 genes easier to observe. These cells recapitulate VSMC behavior by upregulating contractile markers under serum deprivation (19). Under reduced serum conditions and at low confluence, iPASMCs expressed contractile markers (TAGLN, CNN1, ACTA2), but did not express detectable levels of COL1. They responded to TGF-β, in a dose-dependent fashion, by further upregulating the contractile markers and switching on COL1 expression, albeit at a very low level. Forced constitutive expression of NKX2-5 in these cells (iPASMC-NKX) with a lentiviral vector altered their behavior dramatically. Expression of TAGLN, CNN1, and ACTA2 was effectively abolished in iPASMC-NKX, while COL1 was strongly upregulated. The iPASMC-NKX cells still responded to TGF-β by increasing the expression of contractile markers and COL1, but the overall expression of contractile markers remained very low relative to the iPASMC control cells transduced with a control virus. COL1 expression in the iPASMC-NKX on the other hand increased substantially and was much more responsive to TGF-β even at low concentrations. The clear implication is that NKX2-5 does not block TGF-β signaling. Instead, it exerts direct control on the overall expression level of the contractile markers and COL1, effectively overriding TGF-β signaling.

We were able to recapitulate these results in primary cells. We transiently expressed NKX2-5 with a lentiviral vector in primary HPASMCs and cultured them in 10% or 1% serum for 7 days (Figure 4B). Cells treated with a control lentivirus (ShC) upregulated CNN1, TAGLN, and ACTA2 under low-serum conditions. In contrast, contractile marker expression in cells treated with the NKX2-5 lentivirus did not respond to serum deprivation. These results suggest that NKX2-5 exerts direct control on contractile marker expression that can override external signals.

### NKX2-5 regulates contraction, migration, and proliferation.

We tested the function of NKX2-5 in the various cellular processes that characterize the VSMC phenotype. In vitro assays were used to assess cell contraction, migration, and proliferation in the presence or absence of NKX2-5.

**Figure 5:**
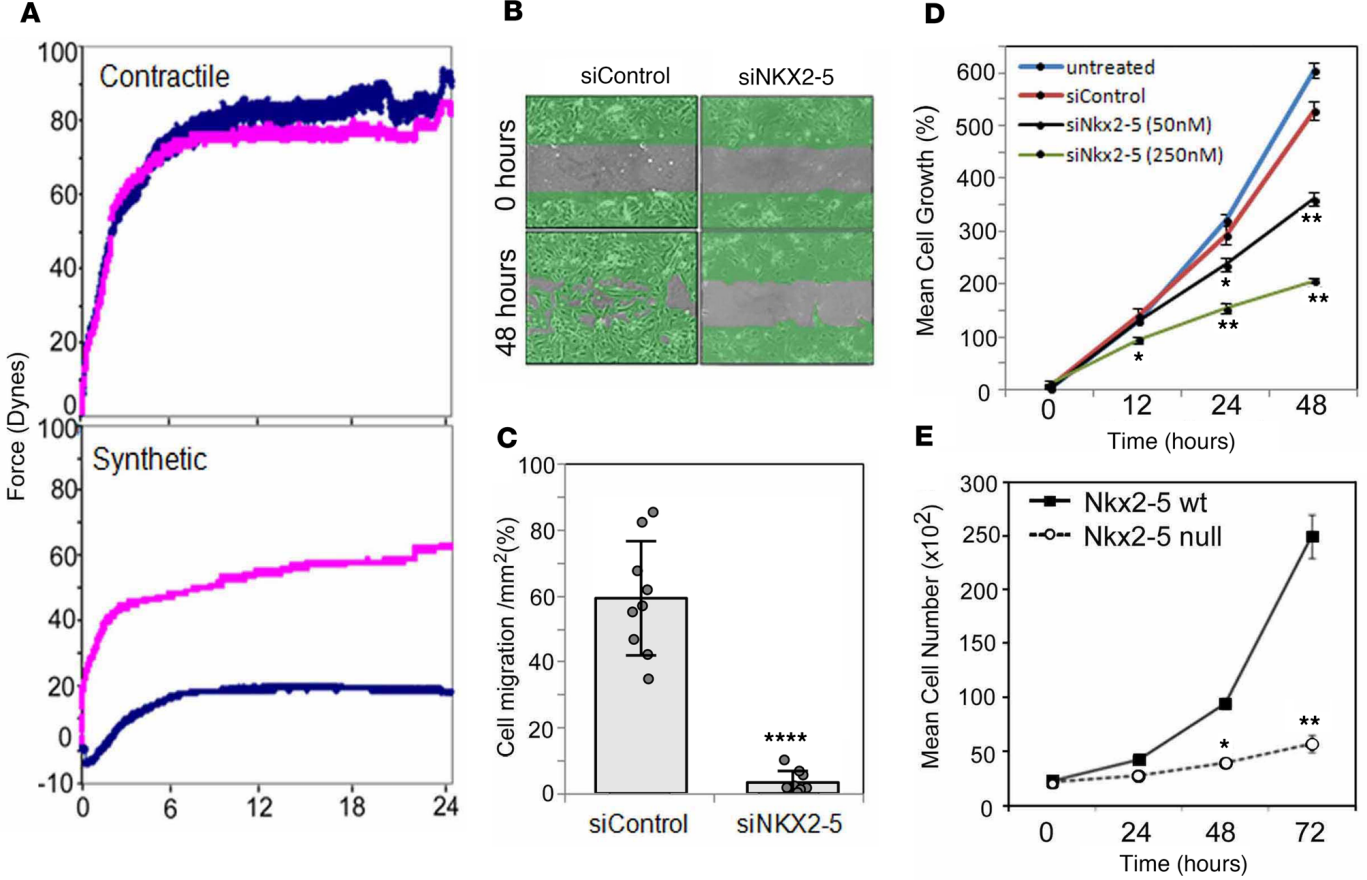
NKX2-5 inhibits VSMC contraction and promotes migration and proliferation. (**A**–**D**) NKX2-5 knockdown was accomplished with siRNA in contractile (1% FBS) or synthetic (10% FBS) HPASMCs. Scrambled oligonucleotides were used as control (siCONTROL). (**A**) Force of contraction exerted on a 3-dimentional collagen gel seeded with treated HPASMCs was measured continuously using a tensioning culture force monitor over 24 hours. siNKX2-5–treated synthetic HPASMCs (red) augmented contractility compared with siCONTROL-treated synthetic HPASMCs (blue). (**B**) NKX2-5 knockdown in synthetic HPASMCs inhibited cell migration into a scratch wound over 48 hours compared with control. (**C**) Nine separate scratch wound experiments were quantified for cell migration into the scratch. ****P < 0.0001 by paired, 2-tailed Student’s *t* test. Data presented as mean ± SD. (**D**) Proliferation of synthetic HPASMCs over 48 hours was measured after no treatment (blue), siCONTROL treatment (red), siNKX2-5 (50 nM, black), or siNKX2-5 (250 nM, green). Statistical significance was confirmed at *P* < 0.05 with 1-way ANOVA. *P < 0.05, **P < 0.01 by post hoc paired, 2-tailed Student’s *t* test. Data presented as mean ± SEM. (**E**) NKX2-5 was deleted in mouse synthetic aortic SMCs (explanted from NKX2-5^flox^ *Col1a2*^CreERT^ mice, *n* = 3 — see Methods) by infection with either Ad.CRE or Ad.GFP (control) (MOI 100). Cell proliferation was measured in triplicate for each cell line over 72 hours. Statistical significance at *P* < 0.05 was confirmed by 1-way ANOVA. *P < 0.05 **P < 0.01 by post hoc unpaired, 2-tailed Student’s *t* test. Data presented as mean ± SEM.

A tensioning culture force monitor (23) was employed to measure the force exerted by VSMCs on a 3-dimensional collagen gel matrix, as a measure of cell contraction. Collagen gels were seeded with contractile or synthetic VSMCs that had been treated either with siCONTROL or siNKX2-5 (Figure 5A) and the exerted force was measured continuously for 24 hours. As expected, we found no appreciable difference between contractile cells treated with siCONTROL and siNKX2-5, consistent with a lack of NKX2-5 expression (Figure 5A). In contrast, siCONTROL-treated synthetic cells exerted markedly lower force (less than 50%) on the matrix compared with siNKX2-5–treated synthetic or contractile cells (Figure 5A). Thus NKX2-5 inhibition rescued the decreased contractility associated with the synthetic phenotype.

We also tested the migratory capacity of VSMCs in the presence or absence of endogenous NKX2-5 using an in vitro scratch wound assay. After 48 hours, synthetic cells showed considerably greater migration than contractile cells. NKX2-5 knockdown significantly inhibited synthetic VSMC migration (Figure 5, B and C). In contractile cells, siRNA treatment had no significant effect (data not shown).

Finally, we assessed the effect of NKX2-5 inhibition on VSMC proliferation. Using siNKX2-5 or siCONTROL oligonucleotides in synthetic VSMCs, we measured proliferation rates over 48 hours. Once again, siCONTROL treatment produced no significant difference compared to untreated, but siNKX2-5 reduced the proliferation rate in a dose-dependent fashion (Figure 5D). These findings were confirmed in synthetic VSMCs explanted from inducible knockout NKX2-5 (NKX2-5^flox^ *Col1a2*^CreERT^) mice, and then exposed to adenovirus particles containing either Cre recombinase (Ad.CRE) or green fluorescent protein (GFP; control). The proliferation rate of cells lacking NKX2-5 was significantly decreased throughout the time course (Figure 5E).

### Mechanism(s) of NKX2-5 activation in VSMCs.

**Figure 6:**
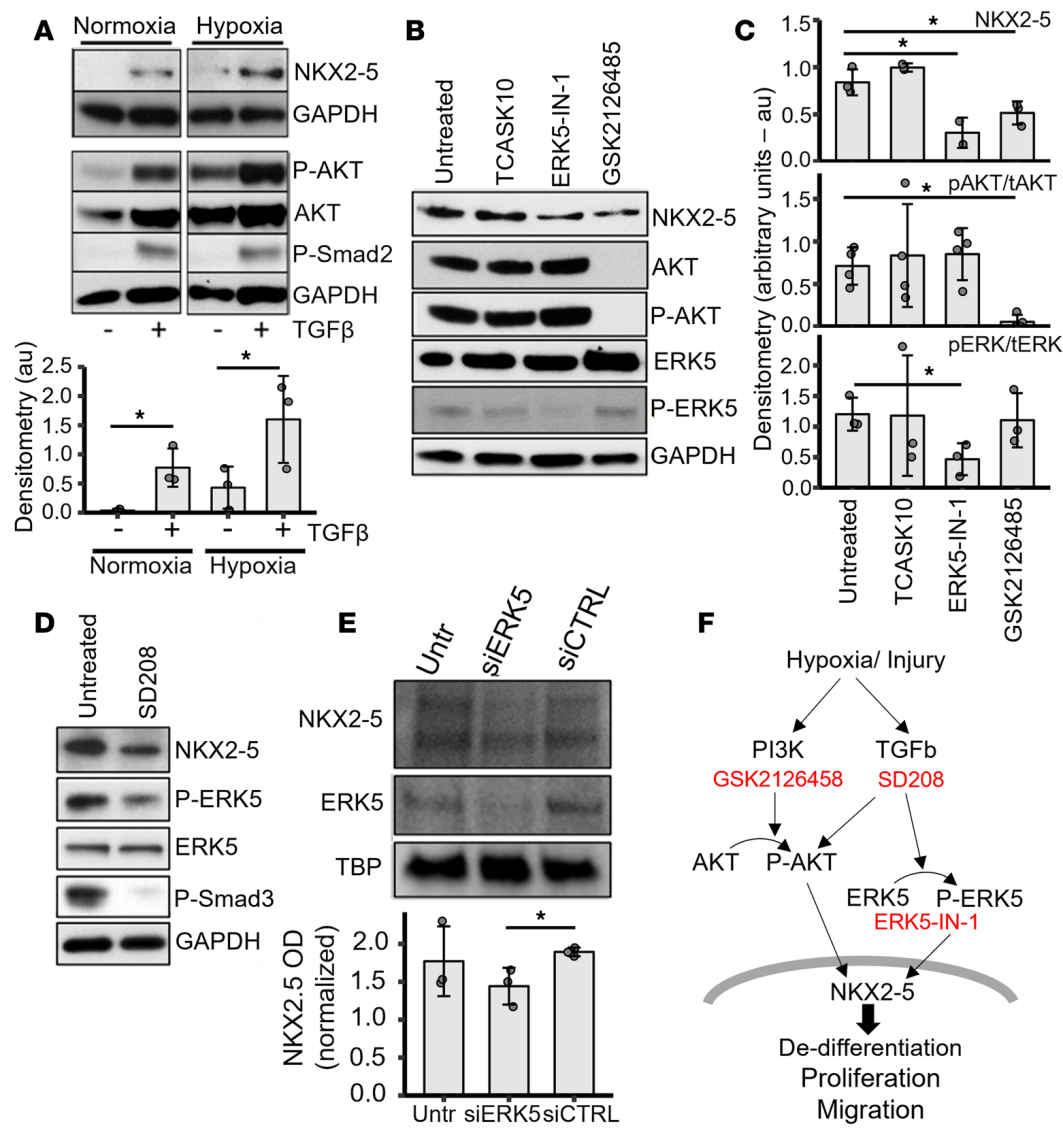
Signaling pathways that result in NKX2-5 activation. (**A**) HPASMCs cultured for 2 days, in synthetic conditions (10% FBS, low density), in hypoxia (1% oxygen), or normoxia, with or without TGF-β1 (4 ng/mL). Total NKX2-5 protein was analyzed by densitometry (*n* = 3). au, arbitrary units. (**B**) Synthetic HPASMCs expressing NKX2-5 were treated with inhibitors for ASK1 (TS ASK10), ERK5 (ERK5-IN-1), and PI3K (GSK2126458). (**C**) Densitometry quantification (*n* = 3) for **B**. (**D**) HPASMCs grown under synthetic conditions, treated with a TGF-β receptor II inhibitor (SD208). (**E**) Western blotting was performed using nuclear extracts from HPASMCs grown under synthetic conditions and treated with vehicle, siRNA (50 nM final concentration) against ERK5 (siERK5), or control siRNA (siCTRL) and quantified by densitometry (*n* = 3). Data presented as mean ± SD. For all blots with multiple proteins, the same samples were run on different, but concurrent, Western blots. (**F**) Potential signaling mechanism for the activation of NKX2-5 in HASMCs: Combinations of hypoxia and injury aided by TGF-β reactivate NKX2-5 via the PI3K and ERK5 pathways. Statistical significance was assessed by paired, 2-tailed Student’s *t* test. *P < 0.05.

A biologically relevant level of NKX2-5 expression in HPASMCs was observed only in synthetic conditions, i.e., 10% serum and low confluence. The process takes several days, but hypoxia and stimulation with exogenous TGF-β can accelerate it, resulting in NKX2-5 appearing earlier than it does with serum alone (Figure 6A). NKX2-5 reactivation coincides with an increase in phosphorylated AKT. To delineate the NKX2-5 activation mechanism(s) in HPASMCs, we investigated signaling pathways downstream of TGF-β and hypoxia. Using specific inhibitors of the PI3K pathway (GSK2126458) (Figure 6, B and C) and TGF-β receptor 1 (SD208) (Figure 6D), we determined that NKX2-5 expression in synthetic HPASMCs is decreased when these pathways are inhibited. Similarly, specific inhibition of extracellular-signal-regulated kinase 5 (ERK5), a MAPK family member associated with cardiovascular development and stress stimuli, results in a significant decrease in NKX2-5 (Figure 6, B and C). Inhibition the TGF-β pathway also resulted in the inhibition of ERK5 phosphorylation to its active form (Figure 6D). Other specific inhibitors for ASK-1 (TCASK10; Figure 6, C and D), TAK1 (also known as MAPKKK; [5*Z*]-7-oxozeaenol), p38 MAPK (SB202190), ERK1/2 (FR 180204), and JNK (BI 78D3) did not have a significant impact on NKX2-5 expression (data not shown). We verified the ERK5 data using siRNA (Figure 6E). An 80% downregulation of ERK5 in cells cultured for 2 days with 10% serum and 0.5 ng/mL TGF-β resulted in a 25% reduction in NKX2-5 relative to cells treated with control siRNA. Our proposed mechanism of NKX2-5 activation in HPASMCs involves the PI3K/AKT, TGF-β, and ERK5 pathways (Figure 6F). We propose that a combination of damage signals, such as hypoxia and the presence of serum combined with TGF-β signaling (either exogenous or autocrine), are needed to reactivate NKX2-5 via the PI3K and ERK5 pathways.

### Targeted NKX2-5 deletion in the chronic hypoxia model of PAH.

Having determined that NKX2-5 is a critical factor in VSMC phenotypic modulation and that hypoxia is one of the main regulatory pathways, we sought to confirm this in vivo using a hypoxia-induced mouse model of PAH. We used an NKX2-5 conditional deletion model to demonstrate that vascular remodeling requires the presence of NKX2-5. The condition for deleting NKX2-5 was activation of the *COL1A2* enhancer, rather than the *COL1A2* promoter. Although a wide variety of cells transcriptionally regulate expression of *COL1A2* via the promoter, activation of the *COL1A2* enhancer is limited to a subset of mesenchymal cells, mostly fibroblasts. In VSMCs, the *COL1A2* enhancer is activated only in cells undergoing dedifferentiation toward a synthetic phenotype driving the overexpression of COL1 (18). Therefore, conditional deletion of genes using the *COL1A2* enhancer to drive Cre recombinase will only be achieved in VSMCs undergoing phenotypic switching to the synthetic phenotype. Contractile VSMCs will not be affected, and for nonvascular cells that do not express NKX2-5, the deletion will have no impact. Cells such as cardiomyocytes, which express NKX2-5 but the *COL1A2* enhancer is not activated, will not conditionally delete NKX2-5. This system ensures that the observed effects are directly attributable to deletion of NKX2-5 from VSMCs.

**Figure 7:**
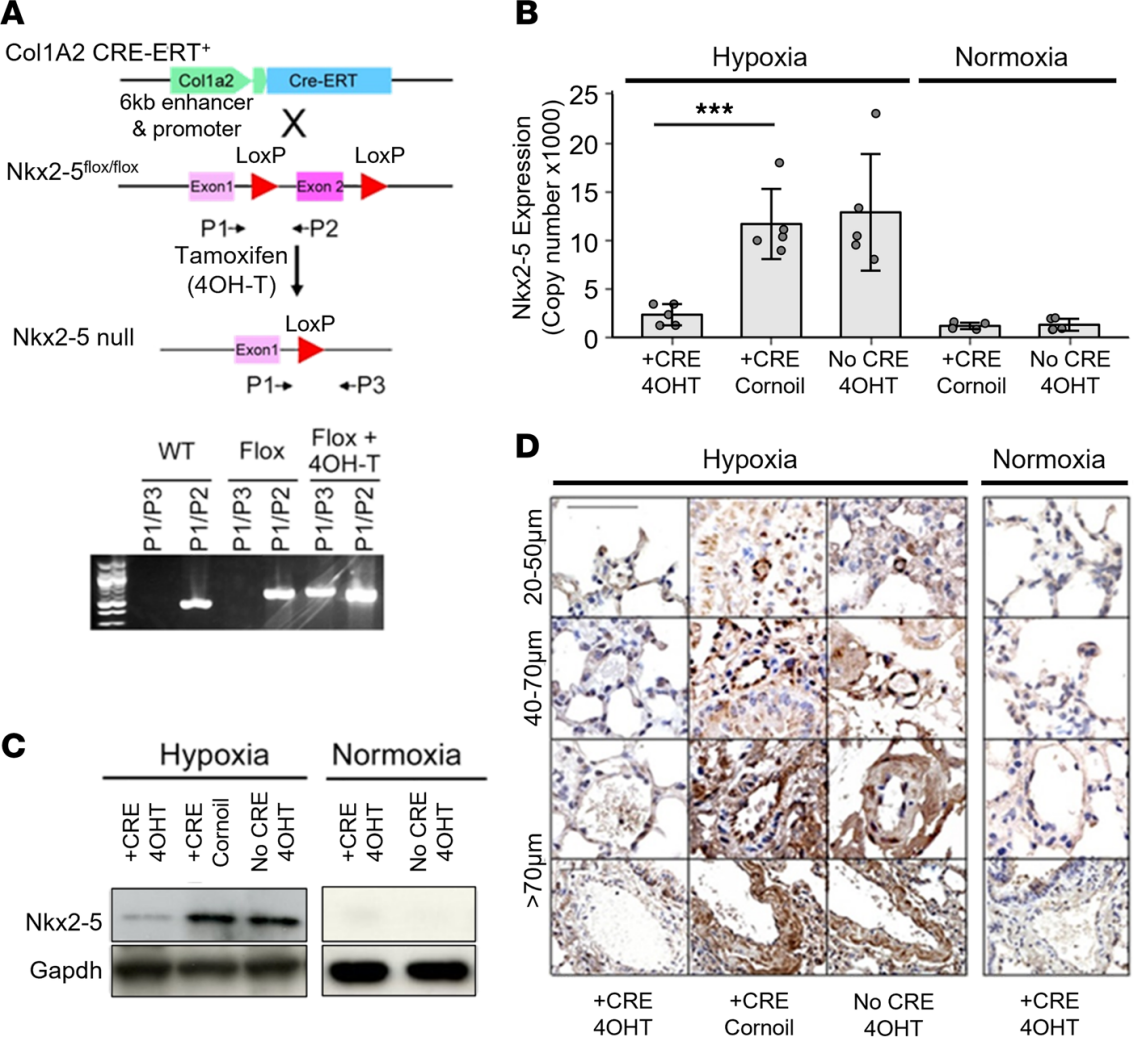
Targeted Nkx2-5 deletion in the chronic hypoxia model of pulmonary hypertension. (**A**) Strategy for conditional deletion of Nkx2-5 in collagen-producing cells using the *Col1a2* enhancer to drive expression of Cre recombinase upon administration of tamoxifen (4OH-T) in adult male mice in the chronic hypoxia model of pulmonary hypertension (*Col1a2*-Cre-ER). PCR primers (P1, P2, and P3) were designed to detect Nkx2-5 deletion as described in the Methods. (**B**) Nkx2-5 mRNA levels in pulmonary arteries of mice (*n* = 8 per group) under hypoxia or normoxia for 21 days. ***P < 0.001 by unpaired, 2-tailed Student’s *t* test. Data presented as ± SD. (**C**) Nkx2-5 protein levels were analyzed by SDS-PAGE in total lung lysates from mice in hypoxia or normoxia. The samples from the hypoxia experiment to determine Nkx2-5 and GAPDH were run on different, but concurrent, Western blots. (**D**) Expression of Nkx2-5 (brown DAB staining) in lung vessels in Nkx2-5–null mouse and control groups were determined via immunohistochemistry under normoxia and hypoxia. Representative images of small (20–50 μm), medium (40–70 μm), and large arteries (>70 μm) are shown. Scale bar: 100 μm.

Inducible conditional NKX2-5–null mice were created by mating NKX2-5^flox^ mice (24) with *Col1a2* enhancer–driven CreERT mice. These transgenic mice contain the mesenchymal cell–specific enhancer of the mouse *Col1a2* gene, driving the expression of a polypeptide consisting of a fusion between Cre recombinase and a mutant ligand-binding domain of the estrogen receptor (25) (Figure 7A). In these *Col1a2*-CreERT–transgenic mice, administration of tamoxifen (4OH-T) activates Cre recombinase in cells where collagen type Iα2 is being overexpressed by VSMCs, including in injured blood vessels. Polymerase chain reaction (PCR) assays using specific primers (P1-P2 and P1-P3) were used to confirm NKX2-5 deletion. NKX2-5 was knocked out in adult NKX2-5^flox^ *Col1a2*-CreERT^+^ (NKX2-5^flox^ Cre^+^) mice in remodeling vessels (Figure 7, A–D) by 4OH-T administration for 5 consecutive days beginning on day 3 after the mice were placed in the hypoxic chamber. *Col1a2* enhancer reporter mice were used to determine the optimal time point of activation of the *Col1a2* enhancer by 4OH-T administration (Supplemental Figure 5). Two groups of controls were used: NKX2-5^flox^ Cre^+^ mice injected with corn oil instead of 4OH-T (NKX2-5^flox^ Cre^+^ Cornoil) and NKX2-5^flox^ Cre^–^ mice injected with 4OH-T (NKX2-5^flox^ Cre^–^ 4OH-T). The mice were sacrificed after 21 days under hypoxia. Parallel groups of mice treated identically were kept under normoxic conditions. Total RNA expression was measured using quantitative PCR (qPCR) (Figure 7B). Pulmonary arteries were analyzed for NKX2-5 protein expression by Western blotting (Figure 7C). Finally, immunohistochemistry for NKX2-5 was performed on lung sections from these mice (Figure 7D). NKX2-5 was not detectable by any of these methods in pulmonary vessels under normoxic conditions; it was elevated in both the hypoxic control mouse groups but was not detectable in NKX2-5–null mice under hypoxic conditions.

### NKX2-5 deletion inhibits vascular remodeling and decreases pulmonary vascular resistance.

**Figure 8:**
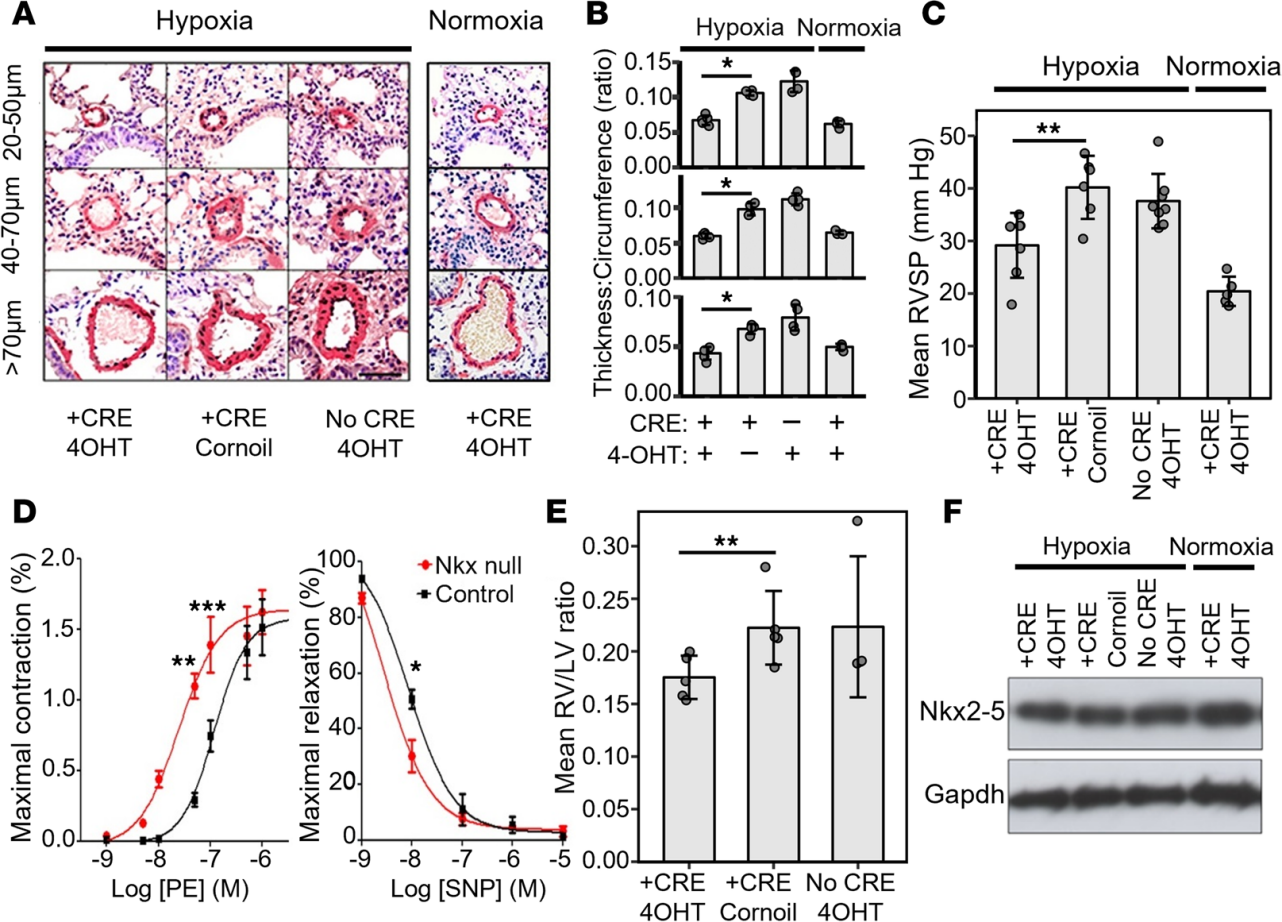
Nkx2-5 deletion ameliorates symptoms of chronic hypoxia–induced pulmonary vascular remodeling. Nkx2-5–null (Nkx2-5^flox^ Cre^+^ 4OH-T) or control male mice were exposed to hypoxia or kept under normoxia for 21 days (see Figure 7 and Methods). (**A**) Sections of the entire left lobe of the mouse lungs were immunostained for ACTA2 to visualize vessels. Representative images of small (20–50 μm), medium (40–70 μm), and large arteries (>70 μm) are shown. Scale bar: 100 μm. (**B**) Muscularization (thickness/circumference ratio) of the arterial wall of pulmonary vessels was analyzed (*n* = 5 mice per group and 300–500 vessels per group) and quantified under hypoxia and normoxia. *P < 0.05 by unpaired, 2-tailed Student’s *t* test. Data presented as mean ± SD. (**C**) RSVP measurements in Nkx2-5–null and control mouse groups under hypoxia and normoxia (*n* = 8 mice per group). **P < 0.01 by unpaired, 2-tailed Student’s *t* test. Data presented as mean ± SD. (**D**) Cumulative concentration-response curves to phenylephrine (PE) or sodium nitroprusside (SNP) in endothelium-intact first- and second-order pulmonary artery segments from Nkx2-5–null (red, *n* = 4) or control (black, *n* = 5) mice. Contraction in response to PE concentration is expressed as mean percentage ± SEM. Relaxation is expressed as mean ± SEM percentage reversal of PE-induced tone. Statistical significance at *P* < 0.05 was confirmed by 1-way ANOVA. *P < 0.05, **P < 0.01, ***P < 0.001 by post hoc unpaired, 2-tailed Student’s *t* test. (**E**) Mean RV/LV ratio as a standard measure of RV hypertrophy. **P < 0.01 by unpaired, 2-tailed Student’s *t* test. (**F**) Expression of Nkx2-5 in whole heart tissue. Representative blot from 3 different experiments. No statistically significant differences observed. Nkx2-5 and Gapdh were run on different, but concurrent, blots.

Medial thickening is one of the characteristics of pulmonary arterial remodeling. This is driven by the expansion of the VSMC layer. The major source of new VSMCs results from the process of phenotypic modulation. The VSMC expansion leads to increased artery muscularization, stronger vascular tone, and narrowing of the vessel lumen. We expect that deletion of NKX2-5 will block this process by preventing VSMCs from switching to the synthetic phenotype and proliferating. We also expect that NKX2-5 deletion will leave ACTA2 staining unchanged, as expansion of the VSMC layer will be prevented. In contrast, in the WT mouse where NKX2-5 can be reactivated, increased muscularization will lead to the appearance of a thicker, more densely stained VSMC layer. Muscularization of small (20–50 μm diameter), medium (40–70 μm), and large (>70 μm) arteries occurred after 21 days under hypoxic conditions in both control mouse groups. The NKX2-5–null mice under hypoxic conditions showed significantly less vascular muscularization (*P* < 0.001) in small, medium, and large vessels, so that they resembled the pulmonary vessels of mice kept under normoxic conditions (Figure 8, A and B). After 21 days of hypoxia, the mice were anesthetized, and the RV systolic pressure (RVSP) was measured using a catheter. Under normoxic conditions, the mean RVSP was 20 ± 1.4 mmHg. After hypoxia, the RVSP in the NKX2-5^flox^ Cre^+^ Cornoil and NKX2-5^flox^ Cre^–^ 4OH-T control groups were elevated to 40.2 ± 2.4 mmHg and 37.6 ± 1.8 mmHg, respectively. However, the RVSP of the NKX2-5–null group was significantly lower (29.2 ± 2.3 mmHg, *P* < 0.01; Figure 8C). There were no differences in mean arterial blood pressure (MABP) between the groups (data not shown).

The impact of NKX2-5 deletion on vessel compliance was measured using myography on first- and second-order pulmonary arteries removed after the RVSP measurements had been taken. A small vessel wire myograph was used to study contraction and relaxation responses in terms of changes in isometric tension development (Figure 8D). Phenylephrine (PE; 1 nmol/L to 5 μmol/L) was used to induce vasoconstriction, and the nitric oxide donor sodium nitroprusside (SNP; 1 nmol/L to 10 μmol/L) was used to induce relaxation. The contraction response curves of vessels from NKX2-5–null mice were shifted to the left compared with controls, showing that the vessels were more sensitive to PE. Similarly, vessels from NKX2-5–null mice were more sensitive to the relaxing effects of nitric oxide (Figure 8D).

RV hypertrophy is a well-described characteristic of the chronic hypoxia model of PAH. Conditional NKX2-5 deletion after 21 days of hypoxia reduced RV hypertrophy, as shown by a significant decrease in the RV/left ventricle (LV) wet weight ratio (Figure 8E) and the absolute wet weights of the RVs alone. There were no physiologically relevant changes in LV wet weight across the groups (data not shown). We tested whether this decrease was a direct result of the deletion of NKX2-5 from the cardiac tissues or an indirect consequence of the pulmonary changes described above. Analysis of NKX2-5 protein expression in the heart by SDS-PAGE and Western blotting revealed no perceptible differences between groups, suggesting that NKX2-5 was not deleted in the heart (Figure 8F). Using a reporter gene driven by the *COL1A2* enhancer, we showed that NKX2-5 in the heart is not deleted because the *COL1A2* enhancer is not activated in cardiomyocytes, even under hypoxic conditions, so Cre recombinase is not expressed (Supplemental Figure 5). As a measure of ECM production, we analyzed COL1 expression in lungs from NKX2-5–null and control groups. We found significantly lower levels of COL1 in the NKX2-5–null mouse lungs (Supplemental Figure 6), confirming the role of NKX2-5 in ECM deposition.

## Discussion

Vascular remodeling is a pathophysiological manifestation of many diseases such as atherosclerosis, peripheral artery disease, hypertension, pulmonary hypertension, and restenosis after angioplasty. It is an adaptive response to injury, inflammation, changes in blood flow, tensile stress, and shear stress (26, 27). Vascular remodeling requires VSMC proliferation, which in turn requires that some of the VSMCs switch to the synthetic phenotype. Over time this expansion of the VSMC population drives thickening of the vessel wall. In this study, we provide multiple lines of evidence demonstrating that NKX2-5 is a powerful modulator of the VSMC phenotypic switch.

We demonstrate conclusively that in cultured HPASMCs, the synthetic phenotype requires NKX2-5. Key phenotypic determinants such as components of the contractile machinery, migration, proliferation, and ECM production, are regulated by NKX2-5. Knockdown or forced overexpression of NKX2-5 abrogates VSMC phenotypic plasticity, locking them into the contractile or synthetic phenotype, respectively.

The role of TGF-β in vascular remodeling and fibrosis is paradoxical. There are strong data implicating TGF-β activity in both normal function and disease. Studies like that by Chen et al. (13) offer strong evidence that TGF-β is vital for normal healthy VSMC contractile function, but other studies offer equally strong evidence that TGF-β superfamily signaling promotes disease (28, 29). A particularly salient example is the BMP receptor mutation in idiopathic pulmonary hypertension, which activates TGF-β signaling (30).

Our data are fully consistent with both observations and help unify the field. We show that NKX2-5 expression directly overrides the effects of TGF-β on VSMC phenotype by shutting down the expression of contractile genes and upregulating collagen, without blocking TGF-β signaling. Furthermore, we find that TGF-β can contribute to NKX2-5 expression in vitro. We propose that when NKX2-5 expression is reactivated due to the combined effect of various upstream signals, it exerts a suppressive effect on the expression of the contractile gene set, antagonizing their upregulation by other pathways, including TGF-β. Both the contractile machinery and the matrix proteins in the synthetic set are TGF-β responsive, but normally TGF-β only promotes the contractile phenotype, because without NKX2-5 the synthetic genes are repressed. In the presence of injury or repair signals, however, TGF-β helps reactivate NKX2-5, which in turn lifts the repression of the synthetic gene set, while attenuating the expression of contractile genes. TGF-β can thus promote either the contractile or the synthetic phenotype, depending on whether NKX2-5 is also expressed.

A new report by Chen et al. (31) investigating the role of TGF-β deletion in aortic aneurysms supports our proposal. The authors found that when the mice are exposed to atherosclerotic conditions after conditional deletion of TGF-β receptor 2 (TGFβR2), aortic aneurysms can readily be detected, due to loss of the smooth muscle phenotype. The authors proposed that vascular injury signals in the absence of TGFβR2 signaling result in activation of KLF4 signaling unchecked by SMAD2/3 repression, which in turn, reverts VSMCs back to a mesenchymal stem cell–like phenotype. Thus, signals like vascular injury that promote the synthetic phenotype lead to VSMC dedifferentiation in the absence of sufficient TGF-β signaling, demonstrating that TGF-β is needed for both the contractile and the synthetic phenotype.

Our data show for the first time to our knowledge that ultimately the phenotypically plasticity of VSMCs is controlled by underlying differentiation-regulating transcription factors such as NKX2-5, which then define the exact function of the overlying signaling pathways that regulate vascular function (e.g., TGF-β), drawing a parallel with phenotypic plasticity observed in immune cells. Therefore, we believe that NKX2-5 is not controlled by a single pathway, but is rather a focal point for signaling arising from conditions associated with damage and inflammation. Our hypothesis explains why TGF-β and other members of the TGF-β superfamily can paradoxically contribute to pathogenesis in vascular disease, despite also being essential for normal vascular function.

Our studies of cell signaling underlying NKX2-5 activation in VSMCs revealed that NKX2-5 induction by hypoxia, serum, and growth factor stimulation proceeds via at least 2 signaling cascades: PI3K and ERK5. The PI3K pathway is directly related to the regulation of cell cycle (32) and proliferation and the ERK5 pathway is associated with cardiovascular development (33) and vascular differentiation programs (34). Yet, these 2 pathways are downstream of many different growth factors, including the TGF-β superfamily, PDGF, FGF, and VEGF, which have both important homeostatic functions and are also involved in blood vessel response to injury. This is consistent with our proposal that input for multiple pathways can induce the synthetic phenotype and proliferation by helping upregulate NKX2-5 beyond a threshold level.

We confirmed this hypothesis using samples from patients with PAH. We robustly document reactivation of NKX2-5 in damaged or diseased vascular tissue, whereas in control, healthy samples it is absent. We used a well-defined animal model that deletes NKX2-5 expression whenever the *COL1A2* enhancer is activated. In adult VSMCs, the *COL1A2* enhancer is inactive under normal conditions. Vascular cells, however, do switch on the *COL1A2* enhancer when they reactivate NKX2-5 under disease conditions. This system will therefore delete the NKX2-5 gene specifically in vascular cells, which have switched on NKX2-5 expression and in mesenchymal cells that do not actually express NKX2-5. As a result, NKX2-5 function will be specifically and selectively ablated only in vascular cells undergoing either phenotypic modulation to the synthetic phenotype or endothelial to mesenchymal transition, while leaving NKX2-5 in the heart intact (confirmed in Figure 8F and Supplemental Figure 5). Activated fibroblasts also activate the *COL1A2* enhancer, but in our hands, we have been unable to detect NKX2-5 expression in lung fibroblasts under any conditions, nor were we able to observe NKX2-5 expression in the connective tissue of diseased lungs, human or murine (Figures 1, 7, and 8). Indeed, the current consensus in the field is that NKX2-5 is heart limited after development. Thus, our system will only delete NKX2-5 either in synthetic VSMCs or in mesenchymal cells that do not express NKX2-5 in the first place. NKX2-5 deletion in this fashion profoundly slowed the progression of vascular disease and inhibited pathological changes in mice subjected to chronic hypoxia. Without NKX2-5, any pathology related to the synthetic VSMC phenotype is greatly attenuated or absent.

We sought further confirmation of our hypothesis that vascular injury and TGF-β superfamily signaling combine to reactivate NKX2-5, using the TGFβR2 kinase–deficient model, which recapitulates key pathological features of systemic sclerosis (35–37). In this model, fibroblast-specific expression of a dominant negative, kinase-deficient TGFβR2 variant paradoxically results in constitutive overexpression of TGF-β1 ligand and strongly increased basal TGF-β superfamily signaling, leading to spontaneous development of fibrosis and PAH with age. Previous work by our group revealed that in young mice, before overt PAH and fibrosis develops, an increased thickness-to-circumference ratio is already present, and SU5416-induced vascular injury rapidly precipitates a strong increase in RSVP. We obtained lung sections from transgenic mice with or without SU5416 treatment and the WT controls and stained them for NKX2-5 (Supplemental Figure 7), revealing that NKX2-5 is indeed reactivated in the transgenic mice and its expression further increased by SU5416. This is as predicted by our proposed model (Figure 6F) that stipulated cooperative activation of NKX2-5 by repair signals stemming from vascular injury and TGF-β superfamily signaling. We have thus shown that vascular remodeling is associated with unexpected NKX2-5 reactivation in vascular cells, in 3 separate settings: in vitro in HPASMCs and in 2 separate, established in vivo models of PAH, one driven by hypoxia and the other driven by TGF-β superfamily signaling and vascular injury.

It should be noted that we have left one potentially important signaling component unexplored in this work, namely mechanosensing. In Dritsoula et al. (19), we demonstrated that YAP binds the NKX2-5 promoter/enhancer. Given the pivotal role of YAP in mechanical signal transduction, this suggests that NKX2-5 is a mechanosensitive gene. The obvious implication is that increased mechanical load may be an independent driver of NKX2-5 reactivation and thus in addition to vascular repair, NKX2-5 may also be involved in maladaptive responses to mechanical stimuli.

A very relevant recent report (38) found that NEDD9, an important scaffolding protein that can associate with both SMAD3 and NKX2-5, has its target preference regulated by oxidative stress and hypoxia among other signals, but is independent of TGF-β signaling. Under oxidative stress in pulmonary endothelial and smooth muscle cells, association with SMAD3 is inhibited and association with NKX2-5 is promoted, enhancing NKX2-5 activity. This report offers evidence of an alternative pathway leading from hypoxia and oxidative stress to posttranslational NKX2-5 activation, thus corroborating our hypothesis that multiple signals converge on NKX2-5. The authors also show that pulmonary hypertension and vascular fibrosis are attenuated by inhibition of NEDD9, further confirming our findings that NKX2-5 is a critical regulator of vascular remodeling.

Our study offers an exciting insight into VSMC phenotypic modulation. During embryonic development, vascular cell progenitors are freely able to proliferate as part of the vascular ontogenesis program, but mature differentiated cells in the adult vessels lose this ability. NKX2-5 follows the same pattern. It has an essential role in directing embryonic vessel formation (39), but its expression is shut down in adult vessels. We found that NKX2-5 can be reactivated in adult vessels to partially recapitulate the embryonic differentiation program, enabling vascular repair and regeneration. Thus, phenotypic modulation does not reflect a permanent and pathologic dedifferentiation of vascular cells, but rather a temporary reactivation of proliferation as part of the normal vascular repair process. It is pathogenic only when it becomes chronic and dysregulated.

Typically, tightly regulated developmental pathways are regulated by a combination of positive and negative control. Examples include polycomb (PcG) and Trithorax (TrxG) (40), Groucho and TCF (41), and Sonic Hedgehog (SHH) and Gli3 (42). We have already documented NKX2-5 acting through competition with the repressor ZEB in the regulation of COL1 expression in VSMCs (18). We expect that a counterpart for NKX2-5 that suppresses the synthetic phenotype also exists and controls VMSC plasticity together with NKX2-5. Other transcription factors implicated in VSMC phenotypic modulation such as SRF, KLF4, myocardin, and ZEB are all functionally linked to NKX2-5 (18, 43–47).

We believe that this role of NKX2-5 in repair can be effectively exploited therapeutically. Although inhibition of NKX2-5 in healthy vessels could potentially block vital repair pathways, when NKX2-5 reactivation has already become pathological, such as in pulmonary hypertension and atherosclerosis or following angioplasty and stenting, it may be very beneficial. Furthermore, NKX2.5 may be a critical factor in applications seeking to exploit ex vivo angiogenesis.

Recently, a study using lentiviral induction of NKX2-5 in vivo suggested a protective role for transient endothelial NKX2-5 expression in atherosclerosis (46). After careful reflection, we concluded that this report does not contradict our findings. The authors injected the lentivirus in the blood stream of mice on an atherogenic diet and followed the mice over 21 days. This will result in temporary expression of NKX2-5 in the endothelium and not the underlying VSMC layer. The authors confirmed that forced expression of NKX2-5 in the endothelium did not persist. Yet, the authors also detected NKX2-5 expression in the smooth muscle cells specifically in the atherosclerotic lesions, directly corroborating our findings. This expression is not due to transduction, but due to the damage caused to the vasculature. Unfortunately, they only investigated the effect of NKX2-5 overexpression in smooth muscle cells cultured under conditions promoting the synthetic/proliferative phenotype, without a comparison with cells in the contractile phenotype, so they do observe not observe any phenotypic changes because they have already made the cells synthetic. Their observation of an atheroprotective effect after transient endothelial NKX2-5 expression is consistent with the repair role we have proposed and it is also consistent with the finding by Deng et al. (48) that MEKK3/ERK5 deletion in endothelial cells can promote arterial remodeling. Our model suggests that a temporary, acute induction of NKX2-5 early on during injury will be beneficial by promoting repair, yet without persisting long enough to induce disordered proliferation and vascular dysfunction. Ultimately this report supports, rather than contradicts, our finding that NKX2-5 is essential for vascular repair, and it is only chronic, persistent expression that is pathologic. Crucially, the authors have independently confirmed NKX2-5 reactivation in the vasculature under disease conditions.

In summary, we have conclusively demonstrated for the first time to our knowledge a role for NKX2-5 expression in human vascular pathology. We showed that NKX2-5 regulates VSMC phenotypic modulation, and that its expression results in VSMC proliferation, migration, inhibition of the cellular contractile apparatus, and modulation of the ECM. We propose that NKX2-5 is part of the vascular repair process, but chronic expression promotes vascular remodeling.

## Methods

### Sex as a biological variable

The human samples originated from donors of both sexes. For the mouse experiments, only males were used to avoid potential interference arising from the estrogenic activity of tamoxifen. Although there are known sex differences in hypertension and scleroderma in general, we do not consider sex as an important biological variable in this study. This is because in our in vitro experiments we did not observe any differences between cells from male and female donors with respect to the function of NKX2-5 in regulating smooth muscle phenotypic modulation.

### Human tissue

Pulmonary tissue from patients in our scleroderma cohorts with PAH as well as healthy and atherosclerotic aortas and coronary arteries were obtained postmortem. Carotid tissue was obtained after carotid endarterectomy and femoral arteries were obtained from amputations after chronic limb ischemia. See Supplemental Figure 2.

### Mice

To generate NKX2-5–deficient mice, we obtained mice homozygous for the *NKX2-5*–floxed allele (a gift from Mohammad Pashmforoush, Keck School of Medicine, University of South California) previously described in Pashmforoush et al. (24). Mice homozygous for the floxed NKX2-5 allele were crossed with *Col1a2*-CreERT mice (49) to generate conditionally induced NKX2-5–knockout mice in cells that have an activated *Col1a2* enhancer element and are therefore overexpressing COL1. *Col1a2*-CreERT–transgenic mice carry a 4OH-T–inducible Cre recombinase element under the control of the enhancer/promoter sequence of the pro-α2(I) collagen gene. 4OH-T induction was carried out either by intraperitoneal injection for 5 consecutive days or included in the normal rodent diet at a concentration of 400 mg/kg (Harlan) and administered throughout the duration of the experiment (50, 51). All mice used in this study were derived from 129 mice and crossed into a C57BL/6 background, and only male mice were used. Genotyping was performed by PCR analysis of tail DNA using Cre-specific primers and primers that spanned intron 1 and exon 2 of the NKX2-5 gene.

The somatic deletion of NKX2-5 in PASMCs after 4OH-T administration was confirmed with PCR using 3 primers: CAAATCTTCGTACTGGAGAGT (P1),CCTGCCTGGAGATTGTACTAGAA (P2), and TGAGCTGAATACATCCCCTAGTTG (P3). The P1-P2 primer pair produces 2 different products: the WT (490 bp) and floxed allele (630 bp). The P1-P3 pair only produces a product with the Cre-deleted floxed allele (~700 bp). The PCR reactions were carried out using the Fast Cycling PCR kit (QIAGEN), with a 57°C annealing temperature and 1 μM primer concentration.

To characterize *Col1a2* enhancer activity within our injury models, we used the *Col1a2* enhancer reporter mouse (*Col1a2*-LacZ-Tg), which was previously described and characterized (18).

#### Chronic hypoxia mouse model of PAH.

Mice (12-week-old male mice, 20–25 g) undergoing exposure to hypoxic conditions were housed in a normobaric hypoxia chamber (850-NBB Nitrogen Dry Box; Plas-Labs) for 3 weeks. Room air balanced with N_2_ achieved an FiO_2_ value of 0.10. CO_2_-absorbent lime was added to maintain the CO_2_ content at below 1.0%. Gas tension and humidity values were determined daily to ensure optimal conditions. Mice were placed in the hypoxic chamber and administered 4OH-T by intraperitoneal injection for 5 consecutive days starting on day 3. On day 21, the RVSP was measured and the mice were sacrificed. The mice were anesthetized with 1.5% isoflurane and placed in a supine position onto a heating blanket that was thermostatically controlled at 37°C. Firstly, the right jugular vein was isolated, and a Millar SPR-671NR mouse pressure catheter with a diameter of 1.4F was introduced and advanced into the RV to determine RVSP. The MABP was also measured by isolating the left common carotid artery and introducing a pressure catheter. Both RVSP and MABP measurements were recorded into a precalibrated PowerLab System (ADInstruments). The mice were sacrificed by isoflurane aesthetic overdose, whole blood samples and lungs were collected, the hearts were removed, and the weights of the RVs and LVs were recorded. RV hypertrophy was determined and expressed as the ratio of the RV weight divided by the LV plus septum weight: RV/(LV + S). The left lung was then fixed by inflation with 10% formalin in PBS before paraffin embedding and sectioning. The remaining lung tissue, heart, and kidney were dissected and snap frozen in liquid N_2_. Plasma was collected by centrifugation and stored at −80°C.

#### Morphologic analysis.

Transverse formalin-fixed lung sections were immunostained using a specific antibody for α-smooth muscle actin (ACTA2). Pulmonary arterial muscularization is expressed as the thickness-to-circumference ratio. Briefly, vessels of 20–100 μm in diameter were imaged in each group of mice. A total of at least 300–500 vessels per group were quantified.

#### Myography.

Mouse lungs were removed from freshly sacrificed mice into cold (4°C) physiologic salt solution. Under light microscopy, second- and third-order pulmonary arterial branches (internal diameter, ~200 μm) were dissected, and 2-mm-long segments were mounted in a dual-chamber wire myograph (model 510A, Danish MyoTechnology) for isometric tension recording. Vessels were bathed in 10 mL of physiologic salt solution, heated to 37°C, and bubbled continuously with 95% oxygen/5% CO_2_and stretched to 90% of the diameter achieved when under a transmural pressure of 15 mmHg. After 30 minutes of equilibration, an automated normalization procedure was performed to determine the arterial lumen necessary for optimal force generation. Vessels were precontracted using the thromboxane-A_2_ analog U46619 (100 nM) 30 minutes after normalization. Endothelial integrity was confirmed by demonstrating 50% or greater relaxation in response to 10 μM acetylcholine applied at the peak of contraction. Concentration-response curves for contraction to PE (10^–9^ to 10^–4^ M) or relaxation in response to SNP (10^–9^ to 10^–4^ M) were constructed.

### VSMC culture

#### In vitro phenotypic modulation.

VSMCs were cultured using a protocol adapted from one previously described (52). Commercially obtained human VSMCs (C-12521, Promocell) were grown from passage 2 in VSMC-specific medium (Promocell) to confluence. The postconfluent cells were maintained in VSMC medium with 10% FBS, passaging them when they reach 60% confluence. These conditions favor the synthetic/proliferative phenotype. To induce contractile gene expression, the VSMCs were seeded at high density (above 70% confluence) in VSMC-specific medium containing 1%–4% serum, depending on the application (see Results). iPASMCs (T0558, ABM Good) were cultured in the same way.

#### Adenoviral transduction.

Explanted mouse aortic smooth muscle cells were grown in T25 flasks (Corning) to 70% confluence and then transduced at an MOI of 100 with either Ad.CRE (Ad5 serotype adenovirus expressing Cre recombinase from the CMV promoter) or Ad.gfp (Ad5 serotype adenovirus expressing green fluorescent protein from the CMV promoter, negative control). Briefly, the cell monolayer was washed in PBS to remove all serum, incubated for 2 hours with adenoviral particles in a minimal volume of medium without serum, washed twice in PBS to remove any remaining viral particles, and then the media were replaced with normal growth media. After viral transduction, the cells were allowed to recover for 24 hours.

#### Lentiviral transduction.

Lentiviral vectors were produced in HEK293T cells (CRL11268, ATCC) using the psPAX2 (12260, AddGene) and pMD2.G (12259, Addgene) packaging plasmids. The vector genome was provided by either pLenti-GIII-CMV-GFP-2A-Puro (ABM, 318840610395) or the SMARTvector Inducible Lentiviral shRNA plasmid (V35H11252, Dharmacon). The plasmids in a genome/capsid/env ratio of 4:3:1 were transfected into HEK293T cells using the FuGene HD transfection reagent (Promega), using 0.5 μg/cm^2^ of culture area, according to the manufacturer’s instructions. The lipid complexes were formed in Opti-MEM I (Thermo Fisher Scientific). Twenty-four hours after transfection, the media were changed to fresh growth media with 0.1% BSA. The supernatant was harvested every 24 hours for 72 hours. Immediately after each harvest, the media were passed through a 0.45 μm PES filter, aliquoted, and snap frozen. VSMCs at 80% confluence were infected using 0.1 mL of crude viral supernatant per cm^2^ of culture area for 6 hours, twice on consecutive days. The transduced cells were selected with 1 μg/mL puromycin for 3–5 days. Prior to use, the cells were allowed 24 hours in growth media without puromycin.

#### RNA interference.

Human VSMCs were grown under conditions that favored either the contractile or the synthetic phenotype, and then preincubated in Opti-MEM I for 3 hours and transfected with 10 nM siRNA Dharmacon NKX2-5 siGenome Smartpool (siNKX2-5) or Dharmacon scrambled control siRNA (siCONTROL) using Oligofectamine transfection reagent (Invitrogen). The cells were used in contraction, migration, and proliferation assays.

#### Contraction.

Measurement of contractile force generated within a 3-dimensional, tethered floating cell–populated collagen lattice was performed as described previously (18, 51). Briefly, using 1 × 10^6^ cells/mL of collagen gel (First Link), we measured the force generated across the collagen lattice with a culture force monitor (CFM). This instrument measures the minute forces exerted by the HPASMCs within a collagen lattice over 24 hours. In brief, a rectangular smooth muscle cell–seeded collagen gel was cast and floated in medium. The collagen gels were tethered to 2 flotation bars on either side of the long edges, and, in turn attached to a ground point at one end and a force transducer at the other. Cell-generated tensional forces in the collagen gel were detected by the force transducer and logged in a personal computer. Graphical readings were produced every 15 seconds, providing a continuous measurement of force generated.

#### Migration assay.

Contractile and synthetic VSMCs were seeded on plastic in the presence of antiproliferative mitomycin C and treated with either siCONTROL or siNKX2-5 (Dharmacon). Briefly, a scratch wound was made across the plate to remove the cells using a device that allows all the scratches to be identical in dimensions. The cells were allowed to migrate into the wound and after 48 hours the percentage cell migration per mm^2^ was measured as described previously (18).

#### Cell proliferation assay.

Human and mouse VSMCs were plated at a density of 15,000 cells per well in 24-well plates and then serum starved for a further 24 hours. The cells were trypsinized and counted using trypan blue (Sigma-Aldrich) exclusion for 0, 12, 24, 48, and 72 hours (*n* = 4 wells per treatment) and number of viable cells was determined. Each isolate was studied at least twice under each condition, and the mean values were taken from all studies conducted with any one isolate.

### Histology, immunohistochemistry, immunofluorescence, image analysis, and Western blotting

#### Antibodies.

Several different anti–NKX2-5 antibodies were validated and used over the course of each study: sc-14033, sc-12514 (Santa Cruz Biotechnology); ab54557, ab35842 (Abcam); and E1Y8H 8792 (Cell Signaling Technology [CST]). Other antibodies used were against ACTA2 (C6189 and A5691, Merck; M0851, Agilent), MYH11 (ab682, Abcam), SMTN (sc-28562, Santa Cruz Biotechnology), TAGLN (ab28811 and ab14106, Abcam), collagen type I (ab34710, Abcam), CNN1 (17819s, CST), intracellular procollagen type I (ab64409, Abcam), connective tissue growth factor (CTGF)/CCN2 (sc-14939, Santa Cruz Biotechnology), FN1 (610078, BD Biosciences), GAPDH (ab8245, Abcam), and TATA-binding protein (TBP) (8515, CT). In addition, antibodies for p-Smad3 (9520, CST), p-Smad2/3 (ab276140, Abcam), Erk5 (3372S, CST), p-ERK5 (3371, CST), AKT (9272, CST), and p-AKT (Ser473) (9271, CST) were used.

#### Patient tissue samples.

Tissue samples were obtained postmortem from patients in our scleroderma cohort, who had a confirmed PAH diagnosis, defined at right heart catheterization by a mean pulmonary arterial pressure of 25 mmHg or greater.

#### Histology.

Tissue samples were formalin-fixed, paraffin-embedded, and sections (3–5 μm) were cut. Tissue architecture, nonfibrillar collagen, ECM, and elastin were determined using standard staining protocols for hematoxylin and eosin (H&E), Masson’s trichrome staining, elastic–van Gieson, and picrosirius red (PSR). The birefringence of PSR staining visualized under polarized light filters is highly specific for collagen where the larger collagen fibers are bright yellow or orange, and the thinner ones, or newer fibers, are green.

#### Immunohistochemistry.

Immunohistochemistry was performed on formalin-fixed, paraffin-embedded tissue. Endogenous peroxidase was blocked using 3% hydrogen peroxide. Optimally diluted antibodies were used at appropriate concentrations. Specificity of staining was confirmed using isotype-matched IgG control antibodies. NKX2-5 staining was visualized by DAB and ACTA2 staining was visualized using a Red Alkaline Phosphatase Substrate kit (Vector Laboratories), and sections were counterstained with hematoxylin. Sections were examined with an Axioskop Z bright-field microscope (Carl Zeiss) using Axiovision software.

#### Immunofluorescence.

Cells were seeded for staining in chamber slides (BD Biosciences), fixed with cold methanol or 4% paraformaldehyde, and permeabilized with 0.2% Triton X-100 in PBS before blocking with isotype-matched serum for 30 minutes. The cells were then stained for 1 hour with various antibodies at 1:100 dilution, washed, and incubated with the appropriate conjugated secondary antibody (Vector BioLabs) before mounting with Vectastain containing DAPI, and examined with an Axioskop Z fluorescence microscope (Zeiss). *Z* stacking and deconvolution protocols were applied to images of cells to determine nuclear localization.

#### Western blotting.

NuPAGE Bis-Tris kits (GE Healthcare) were used according to the manufacturer’s instructions. MOPS running buffer and nitrocellulose membranes (Hybond-C; GE Healthcare) were used for blotting. Protein bands were visualized using chemiluminescence with HRP-linked secondary antibodies (GE Healthcare) and photographic film (Hyperfilm ECL; GE Healthcare).

### RNA isolation and qRT-PCR analysis

VSMC were lysed in 1 mL QIAzol (QIAGEN), mixed with 200 μL chloroform, centrifuged at 13,000 rpm and 4°C, and the aqueous phase was transferred to fresh tubes and mixed with 1.5 volumes of absolute ethanol. This was then transferred to RNeasy spin columns (QIAGEN) and RNA was purified according to the manufacturer’s instructions. RNA purity and quantity were determined using a NanoDrop spectrophotometer and integrity was determined by capillary electrophoresis (Agilent Bioanalyzer). Samples were used if A_260/280_ was greater than 1.98 and the RIN was greater than 9.5. RNA (1 μg) was reversed transcribed using a Quantitect reverse transcription kit (QIAGEN), including a gDNA wipeout step and RT negative controls for each sample. All primers spanned an intron and potential secondary structure was excluded by M-fold. cDNA was amplified using a Sensimix No SYBR greenPCR kit using a Rotor-Gene 6000 (Corbett Research) and melt curves were checked for product specificity. Standards (10^7^–10^1^ copies/μL) were also prepared from VSMC cDNA purified by agarose gel extraction (QIAGEN) and were included in each run, as were no-template controls to exclude template contamination, and for 2 reference genes (*ACTB* or *SDHA*), RT negative controls to exclude gDNA contamination. Assay efficiency was determined from the standard curves, which were used to derive copy numbers. Assay specificity was confirmed by BLAST, a single peak on melt curves, a single band on agarose gel, and product sequencing. A panel of 6 reference genes was initially used to select the most stable genes for data normalization using geNorm (53). The primer sequences for the human genes of interest (5′–3′) were as follows: NKX2-5, forward GGACCCTAGAGCCGAAAA and reverse CCGCTCCAGCTCATAGA; COL1A2, forward GCACATGCCGTGACTTGAGA and reverse GGATTAGTTCCTACGTGATACCTAC; ACTA2, forward GGAATCCTGTGAAGCAGCTC and reverse CCGATCCACACGGAGTA; TAGLN, forward GATTCTGAGCAAGCTGGTGA and reverse TCTGCTTGAAGACCATGGAG; CCN2, forward GCTGACCTGGAAGAGAACATTA and reverse GCTCGGTATGTCTTCATGCT; SDHA, forward AGAAGCCCTTTGAGGAGCA and reverse CGATCACGGGTCTATATTCCAGA; ACTB, forward CACCATGTACCCTGGCATT and reverse CCGATCCACACGGAGTA.

### Mouse sample protein analysis

Mouse tissues (pulmonary, vascular, and heart) were dissected to isolate VSMCs, which were then homogenized and lysed. The lysates were either stored at −80°C or analyzed by Western blotting as described above.

### Inhibitor treatment

iHPASMCs (ABM) were grown on collagen-coated flasks in DMEM supplemented with 5% serum. The cells were plated to 60%–70% confluence, and then serum starved and treated with 2 ng/mL TGF-β (R&D Systems, 240-B-010) for 24 hours. After TGF-β treatment, the cells were placed in 5%FBS/DMEM and the inhibitors were added as follows: 10 μM TCASK10 (Tocris, 4825), 5 μM ERK5-in-1 (Selleckchem, S7344), and 3 μM GSK2126458 (Selleckchem, S2658). The cells were treated with the inhibitors for 24 hours before harvesting for protein and RNA. Western blot assays were used to assess the levels of the proteins using antibodies against NKX2-5 (ab54567, Abcam) and GAPDH (ab8245, Abcam) from Abcam; AKT (9272, CST), p-AKT (9271, CST), ERK5 (3372, CST), p-ERK5 (3371, CST), p-SMAD2 (3101, CST), and p-SMAD3 (9520, CST). Images were analyzed using ImageJ (NIH).

### Statistics

Data are presented as mean ± SEM or ± SD. Unpaired *t* tests were used for comparison between 2 groups, and 2-way analysis of variance (ANOVA) used for comparison among groups. *P* values of less than 0.05 were considered significant. Data obtained from the 2-way factorial design were analyzed with 2-way ANOVA. For in vivo studies, changes in MABP, RVSP, RV/LV ratio, and muscularization of arteries were analyzed by 1-way ANOVA. Neointimal/lumenal areas of remodeled vessels and concentration-response curves were fitted to all the data using nonlinear regression and the –log(M) (where M is molar concentration) and analyzed using 2-way ANOVA (GraphPad Prism Software). For densitometry analysis of Western blots, where there were 3 or fewer comparisons to be made, statistical significance was assessed with paired, 2-tailed *t* tests. For a single comparison, the significance threshold was set to 0.05, while for 2 or 3 comparisons the significance threshold was set to 0.01 to control the false discovery rate. For experiments with a large number of comparisons, such as Figures 2–4, ANOVA was used with posthoc *t* tests to confirm key results. Since several genes are being examined and we expect that their behavior should be coordinated, in lieu of multiple testing adjustments we impose the stronger condition that differences in all linked genes should be significant at the *P* < 0.05 level in order to reject the null hypothesis. The results of the ANOVA analysis for Figures 2–4 can be found in Supplemental Figures 8–10, respectively.

### Study approval

All human tissue used in this study was obtained with written consent for research purposes and approved by the Royal Free and Medical School local Research Ethics Committee. All animal measurements were carried out under Home Office animal licence no. PPL-70/8050. All animal studies conformed to the UK Animals (Scientific Procedures) Act 1986. Strict adherence to institutional guidelines was practiced, and local ethics committee and Home Office approval were obtained prior to all animal procedures.

### Data availability

The raw data used to make the graphs found in the manuscript’s figures are available in the accompanying Supporting Data Values Excel file.

## Author contributions

IP designed and performed experiments, acquired and processed data, and wrote the manuscript. AD, PK, RSB, SLT, EC, XS, and AJH performed experiments and acquired and processed data. CPD and DJA designed experiments, provided reagents, and edited the manuscript. MP designed and performed experiments, acquired and processed data, and wrote and edited the manuscript.

## Supplementary Material

Supplemental data

Unedited blot and gel images

Supporting data values

## Version 1 04/23/2024

In-Press Preview

## Version 2 05/22/2024

Electronic publication
