# Supplementary material for: NKX2-5 regulates vessel remodeling in scleroderma-associated pulmonary arterial hypertension
Source: JCI Insight. 2024 Apr 23;9(10):e164191. doi: 10.1172/jci.insight.164191 (PMC11141943; doi:10.1172/jci.insight.164191)
Supplement: Supplemental data [file jciinsight-9-164191-s136.pdf]

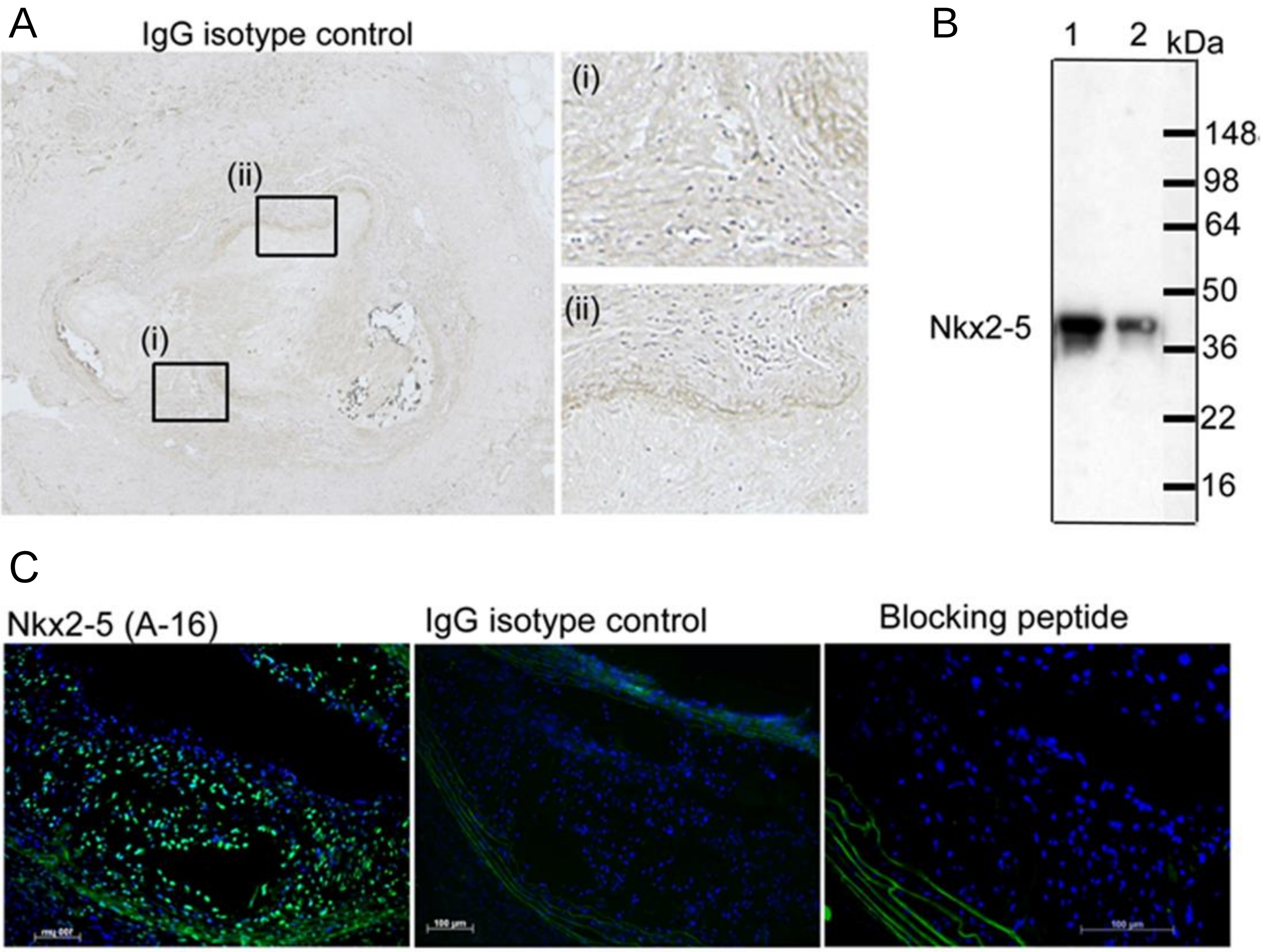

Supplementary Figure 1 Validation of NKX2-5 immunostaining and immunofluorescence. (A) NKX2-5 used in immunostaining experiments was validated by replacing the primary antibody with an isotype-matched antibody in an occluded popliteal artery from a patient with peripheral artery disease. **(B)** The antibody was tested on tissue homogenates from mouse heart (lane 1 antibody cross-reacts with mouse NKX2-5) or atherosclerotic vessels (lane 2). **(C)** Another NKX2-5 antibody (A16,) was tested on human atherosclerotic carotid endarterectomy tissue using immunofluorescence. NKX2-5 was depicted in green (Alexafluor^{488}), nuclei in blue (DAPI). The NKX2-5 nuclear staining seen with the antibody was not observed in the isotype control and could be blocked by a pre-incubating the antibody with a specific blocking peptide, prior to staining.

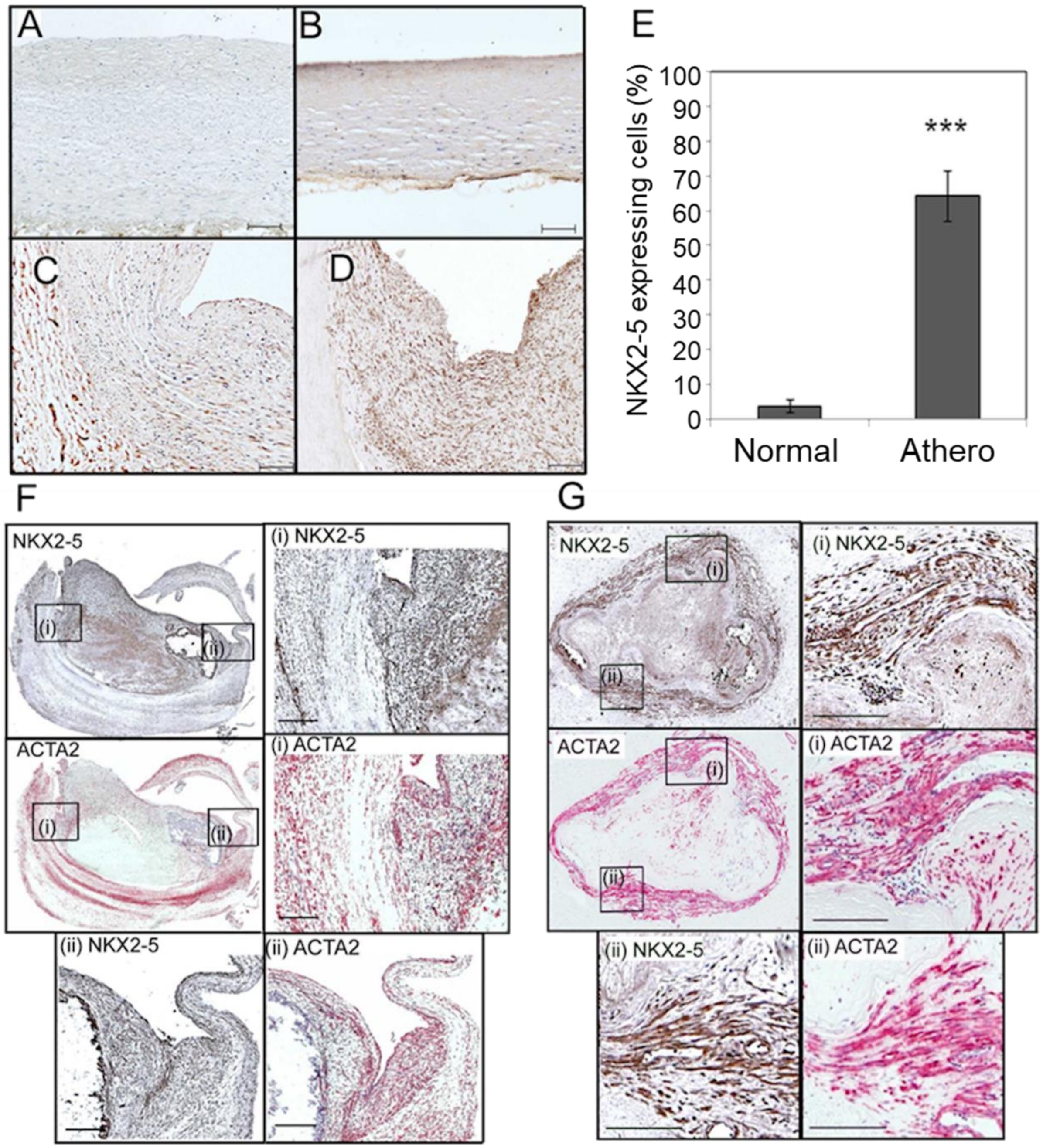

Supplementary Figure 2: Expression of NKX2-5 in human healthy vasculature and in vascular pathology. NKX2-5 expression is absent in healthy vessels as shown in **(A)** a left coronary artery sample and **(B)** carotid artery sample but highly expressed in diseased vessels such as **(C)** a coronary artery and **(D)** a carotid artery atherosclerotic lesions. Sections were immunostained for NKX2-5 (DAB, brown) and counterstained with haematoxylin. Scale bars (100mm). **(E)** The percentage of cells with NKX2-5 staining in sections of atherosclerotic carotid (4 independent fields of view on 3 athero vessels, total n=12,) and healthy (Normal subjects total n=12) tissue. Images were scored by 3 independent blinded observers. The number of NKX2-5+ cells was expressed as a percentage of total cells. *** P<0.001 by paired two-tailed Student's *t* test. Error bars, ± s.e.m. **(F)** Serial sections of tissue taken from carotid endarterectomy (n=3) or **(G)** occluded popliteal artery from a patient with peripheral artery disease (n=3) were stained by immunocytochemistry for NKX2-5 (brown) and ACTA2 (red), revealing co-expression of NKX2-5 in VSMC in both the media and neointima of the vessels. Scale bars 1mm. High magnification is shown in inserts (i) and (ii). Scale bars 200mm.

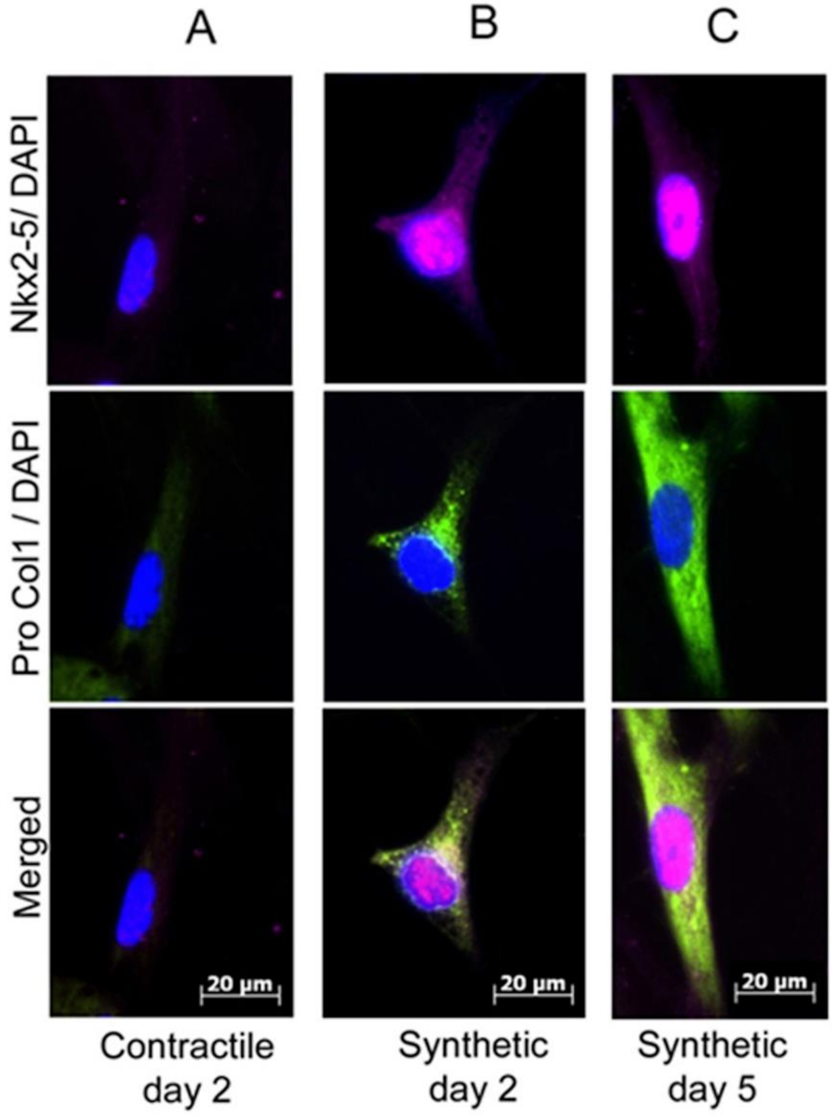

Supplementary Figure 3. Nuclear expression of NKX2-5 in human vascular smooth muscle cells. Human aortic smooth muscle cells were grown in culture under conditions favouring either the contractile (A) or synthetic phenotype (B, C) (see methods and figs 3,4). The cells were fixed and permeabilised and before 3-colour immunofluorescence was carried out using primary antibodies for NKX2-5 (pink, Alexafluor ^{680}), intracellular pro-collagen type I (green, Alexafluor^{488}). DAPI (blue) was used to stain the nuclei. Z-stacking and de-convolution using Axiovision software was applied to determine nuclear staining and the images of each colour with DAPI or merged 3-colors are depicted. Scale bars, 20μm).

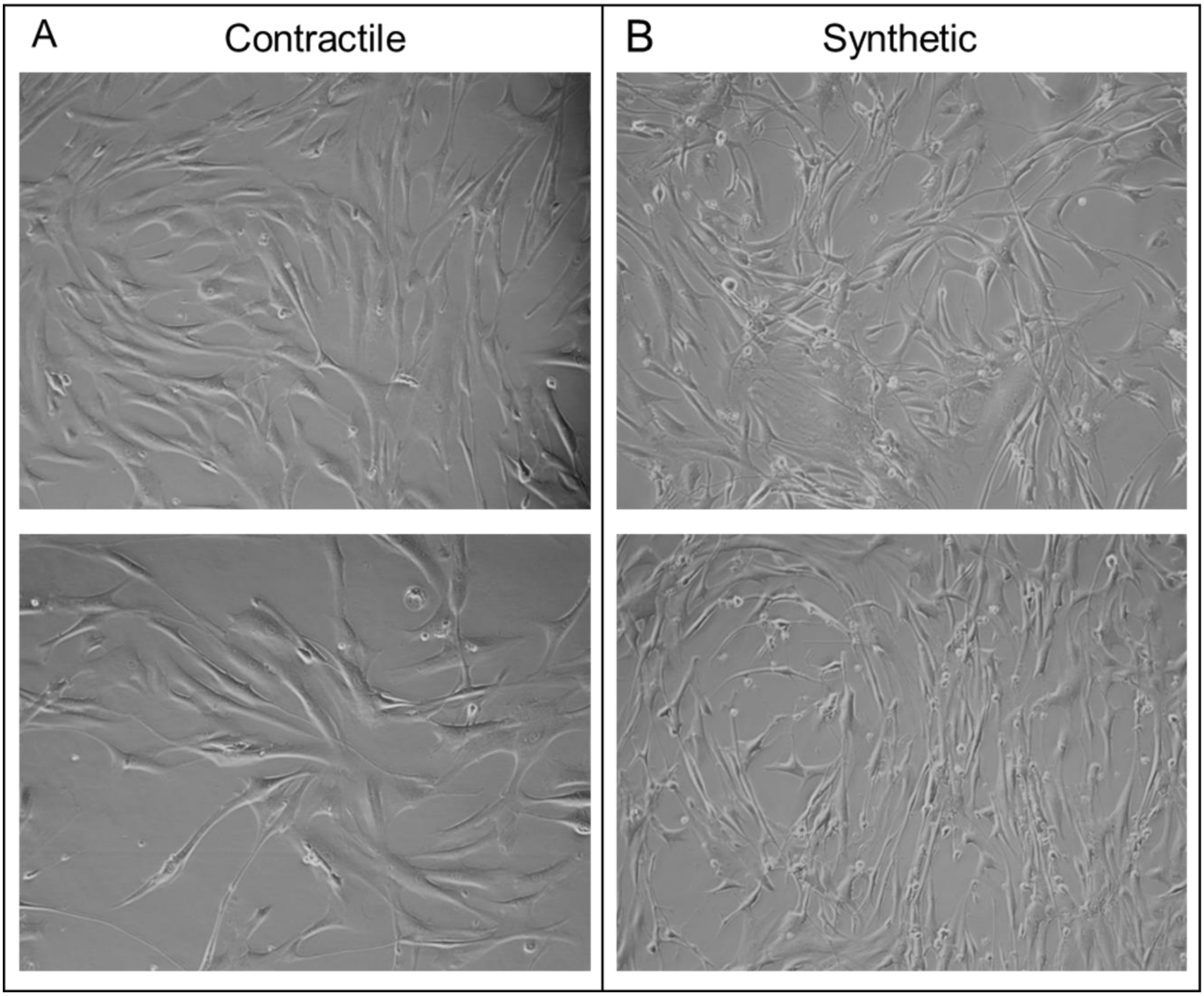

Supplementary Figure 4. Cell morphology of contractile and synthetic cells. Human pulmonary SMC, were cultured under contractile (A) or synthetic conditions (B) and bright field microscopy images of 4-5 different fields were taken to assess cell morphology. Two different optical fields are shown for each culture condition. Culture under synthetic conditions produces a marked change in morphology, the cells become less spread out on the matrix, hence looking thinner and spindlier. They also extend multiple pseudopodia, indicating increased motility and begin to proliferate.

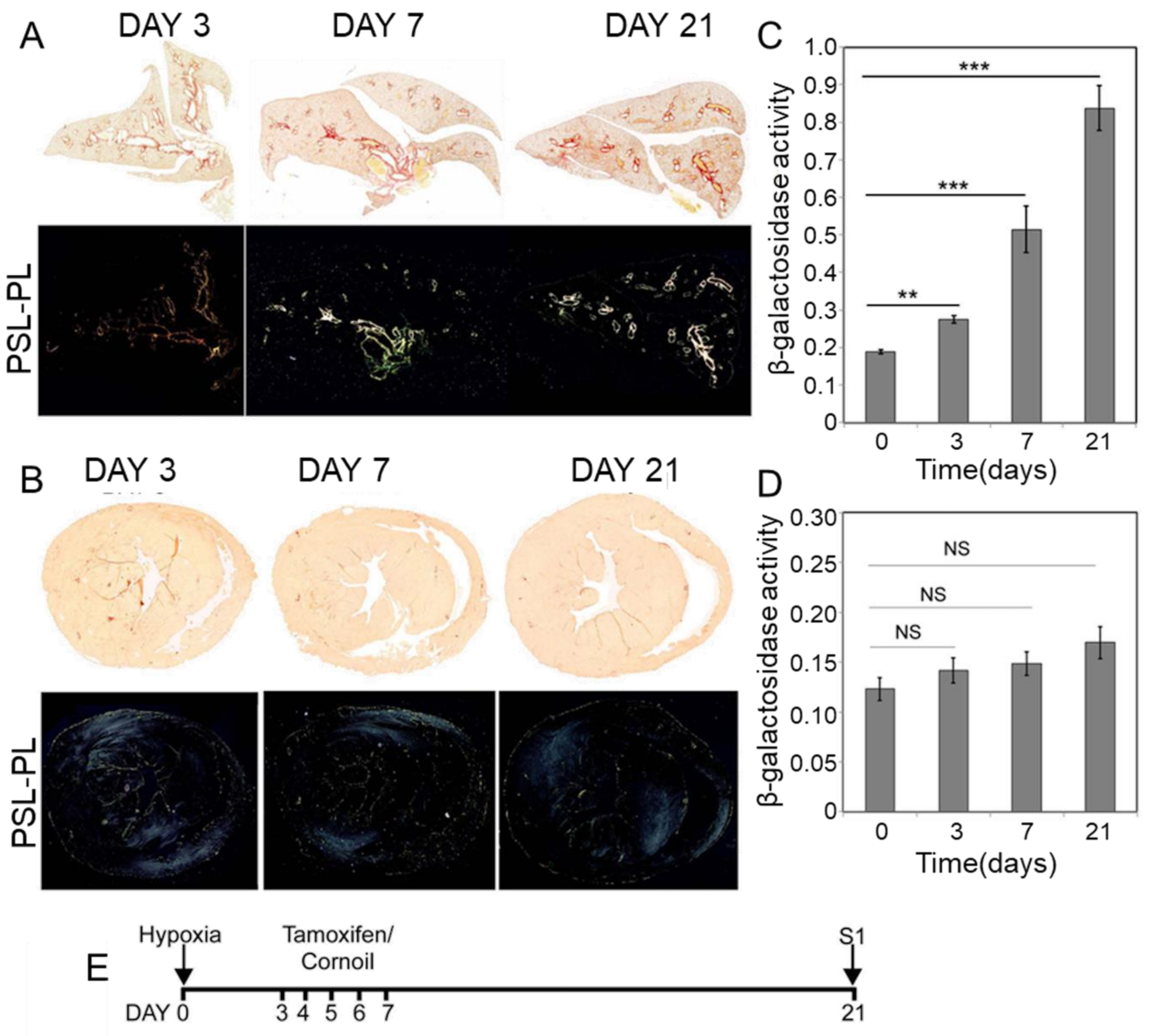

Supplementary Figure 5. Col1a2 enhancer activation in the chronic hypoxia mouse model of Pulmonary Hypertension. Collagen type Ia2 enhancer reporter mice (Col1a2-LacZ-Tg) in which the Col1a2 enhancer is fused to the b-galactosidase gene were used to determine activation of the Col1a2 enhancer at the optimum time point for the administration of tamoxifen in mice placed the hypoxic chamber for a time course of 21 days. Mice were sacrificed at days 3, 7 and 21 days and the lungs **(a)** and hearts **(b)** were analysed for collagen deposition using PSR staining visualized under polarized light (PSR-PL). ** P<0.01, *** P<0.001 by paired two-tailed Student's *t* test. Error bars, ± s.e.m. Specific activity of the b-galactosidase was also determined in lung **(c)** and heart **(d)** tissue lysates. No b-galactosidase activity could be seen in the heart, demonstrating unequivocally that hypoxia does not activate the COL1A2 enhancer in heart tissue by hypoxia within the experimental time-frame. In the lung however perivascular b-galactosidase activity starts to appear at day 3 and becomes stronger at 7 and 21 days. Based on these results it was concluded that the COL1A2 enhancer can drive specific expression in lung blood vessels, but not in the heart, in response to hypoxia. **(e)** Experimental plan for targeted deletion of NKX2-5. NKX2-5^{flox} mice with the tamoxifen-responsive Col1a2CreERT^{+} cassette, were placed in a hypoxic chamber and Cre recombinase expression to delete NKX2-5 was induced by administering tamoxifen intraperitoneally for 5 days (days 3-7). Cornoil was the vehicle control.

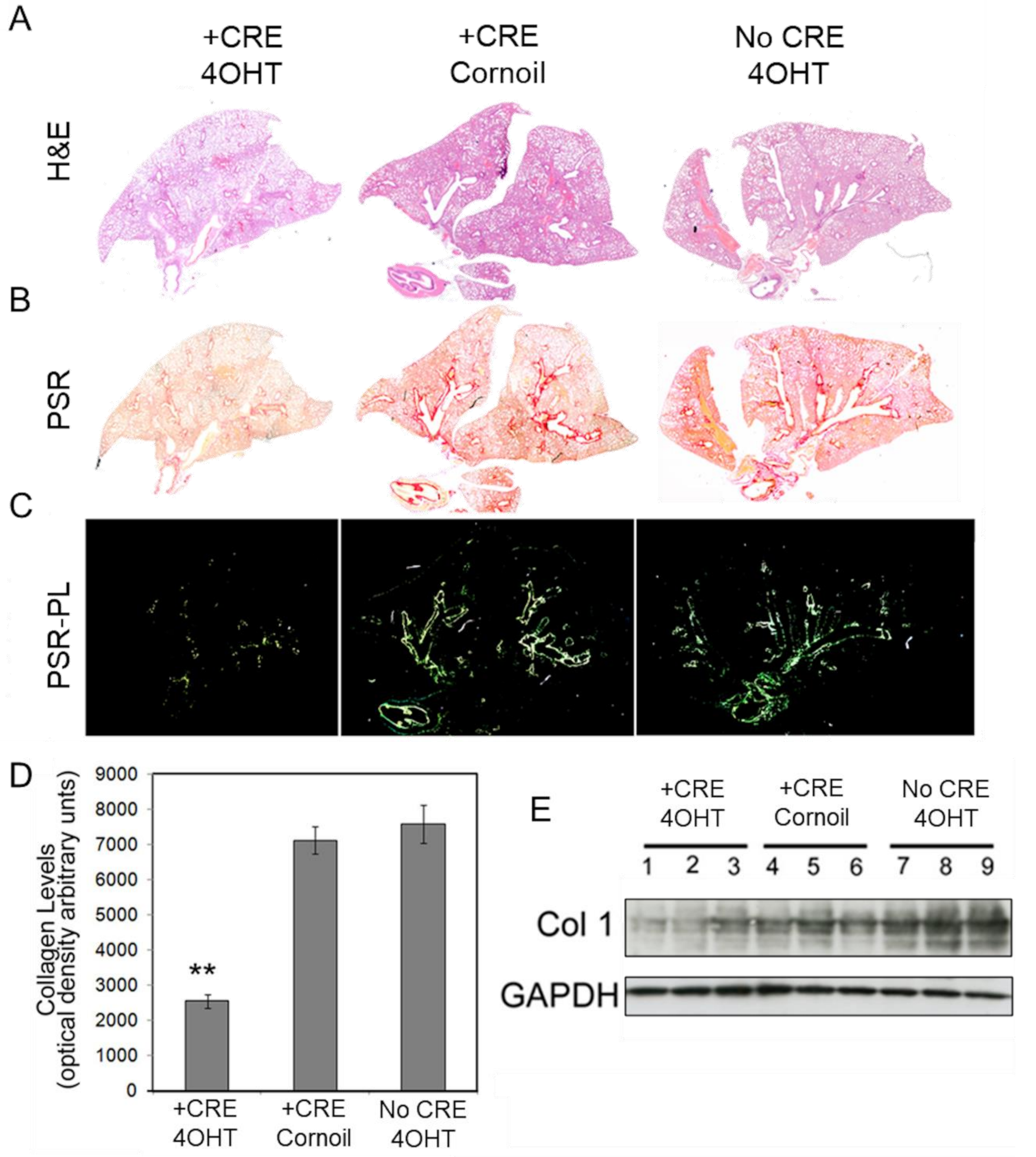

Supplementary Figure 6. Effect of targeted NKX2-5 deletion on pulmonary collagen deposition in the chronic hypoxia mouse model of Pulmonary Hypertension. NKX2-5 null (NKX2-5^{flox} Cre^{+} 4OH-T) or control male mice (NKX2-5^{flox} Cre^{-} 4OH-T and NKX2-5^{flox} Cre^{+} Cornoil) were used in the chronic hypoxia model of pulmonary hypertension or under normoxia for 21days (see fig 7 and suppl fig 5). Sections of the entire left lobe of the mouse lungs were stained with **(A)** haematoxylin and eosin (H & E) to assess pulmonary architecture and **(B)** Picrosirius Red (PSR) for collagen deposition **(C)** visualized under polarized light (PSR-PL). **(D)** Collagen levels were determined by measuring the optical density on five PSR stained sections at four different plains across the lung per mouse (n=5 mice). ** $P<0.05$, *** $P<0.0005$ by paired two-tailed Student's *t* test. Error bars, ± s.e.m. **(E)** Collagen type 1 levels was analysed in whole lung tissue lysates by SDS-PAGE. Lysates from three mice in each group (n=3) are shown and GAPDH was used as loading control.

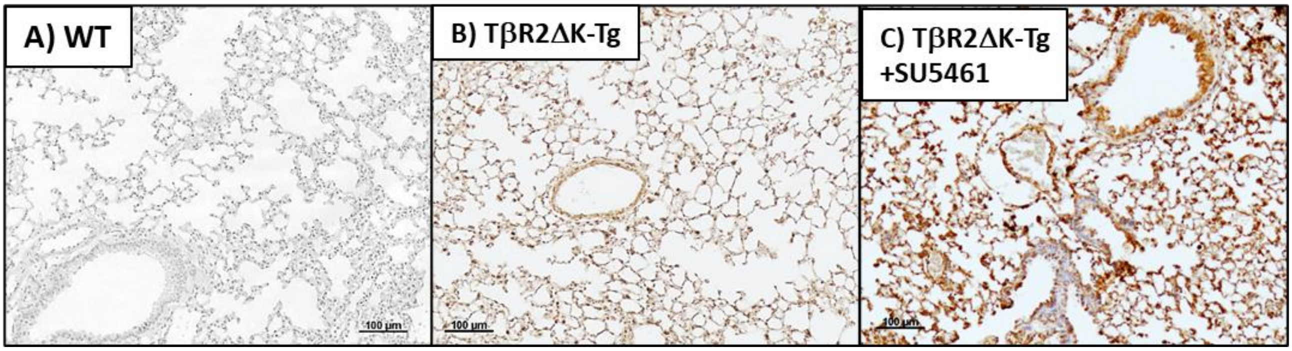

Supplementary Figure 7. Expression of NKX2-5 in the TGFβR2 kinase deficient model of systemic sclerosis. We obtained lung tissue section prepared by Derrett-Smith et al (36), and visualized NKX2-5 by immunostaining (DAB, brown). The samples were from **(A)** WT animals with vehicle, **(B)** Transgenic animals with vehicle and **(C)** Transgenic animals treated with SU5461.

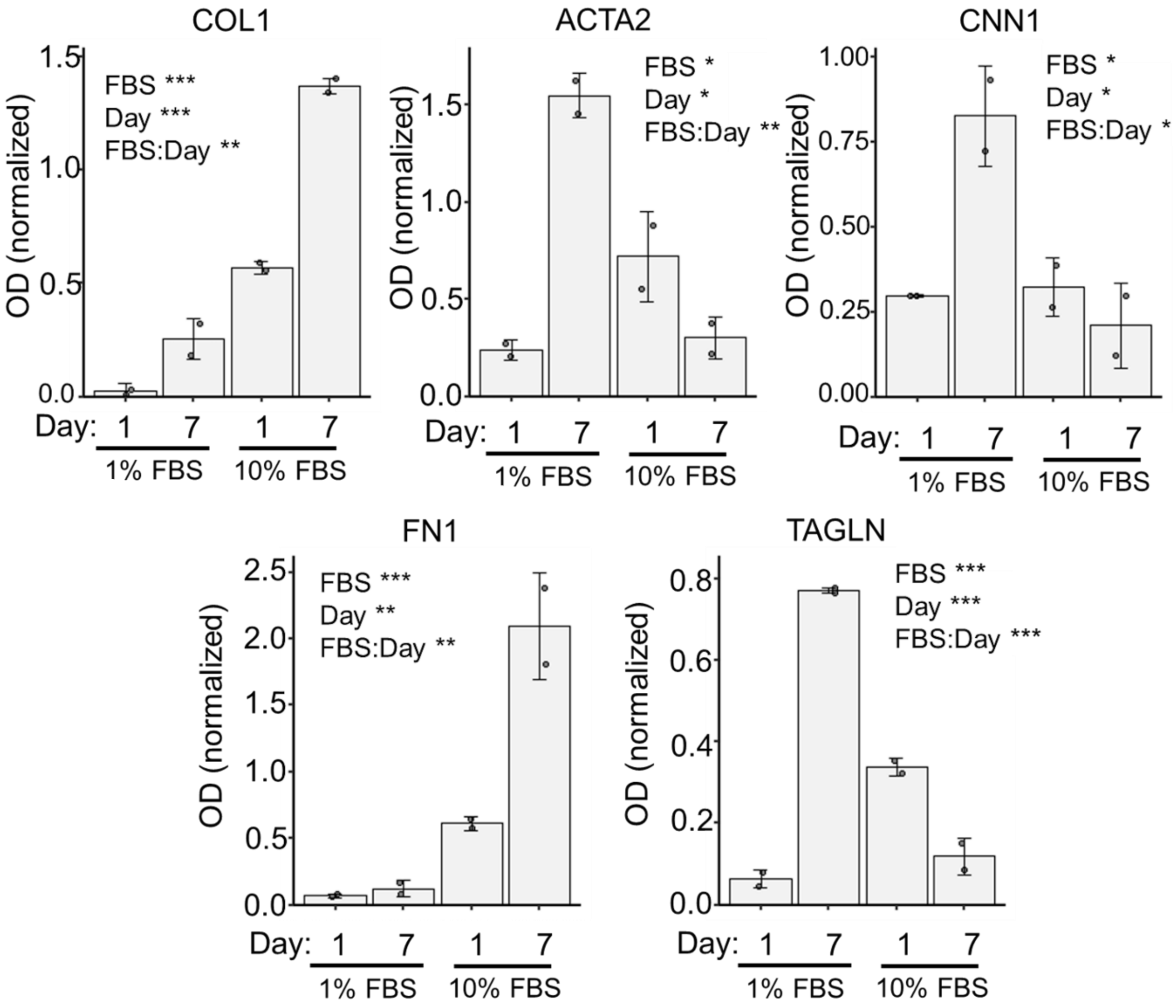

Supplementary Figure 8. Densitometry analysis for figure 2. Two independently obtained western blots from the same samples were analysed by densitometry. Optical density for COL1, FN1, ACTA2, TAGLN and CNN1, was normalized to GAPDH (normalized OD). Statistical analysis was carried out by 2-way ANOVA using FBS and Day as the dependent variables. The statistical significance for each dependent variable and their interaction factor (FBS:Day) is indicated on the graph. *** = $p<0.001$, ** = $p < 0.01$, * = $p < 0.05$

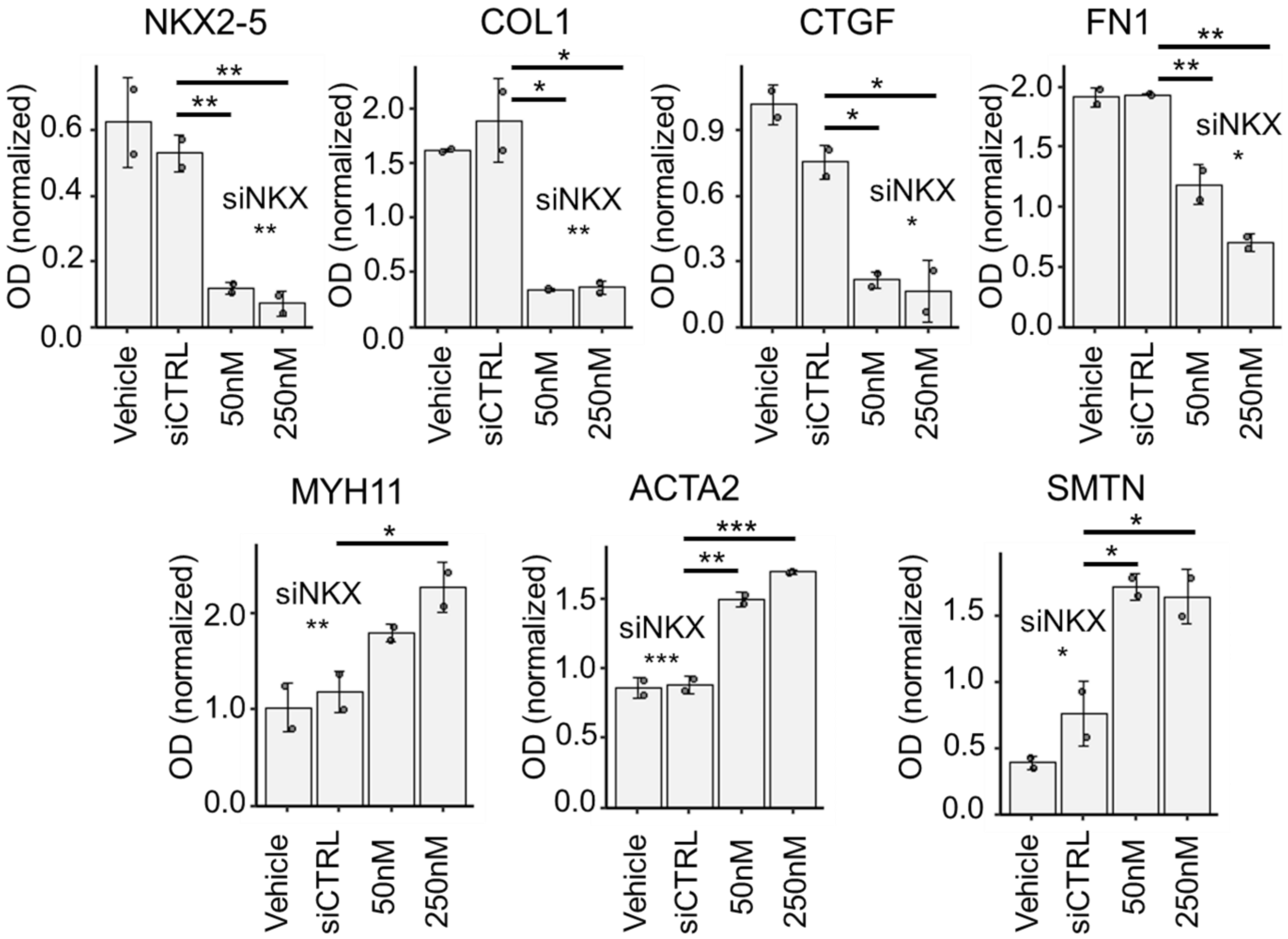

Supplementary Figure 9. Densitometry analysis for figure 3. Two independently obtained western blots from the same samples were analysed by densitometry. Optical density for NKX2-5, COL1, CTGF, FN1, MYH11, ACTA2, and SMTN, was normalized to GAPDH (normalized OD). The amount of siNKX2-5 added is indicicated by either 50nM or 250nM. For the control siRNA (siCTRL) 250nM was used. Statistical analysis was carried out by 1-way ANOVA using the siCTRL and siNKX2-5 samples, using the amount of siNKX2-5 as the dependent variable. The statistical significance is indicated by the label siNKX and a star. Post-hoc comparisons between the siNKX2-5 samples and siCTRL were carried out using the Tukey test. The statistical significance is indicated with a line connecting the two samples compared. *** = $p<0.001$, ** = $p < 0.01$, * = $p < 0.05$

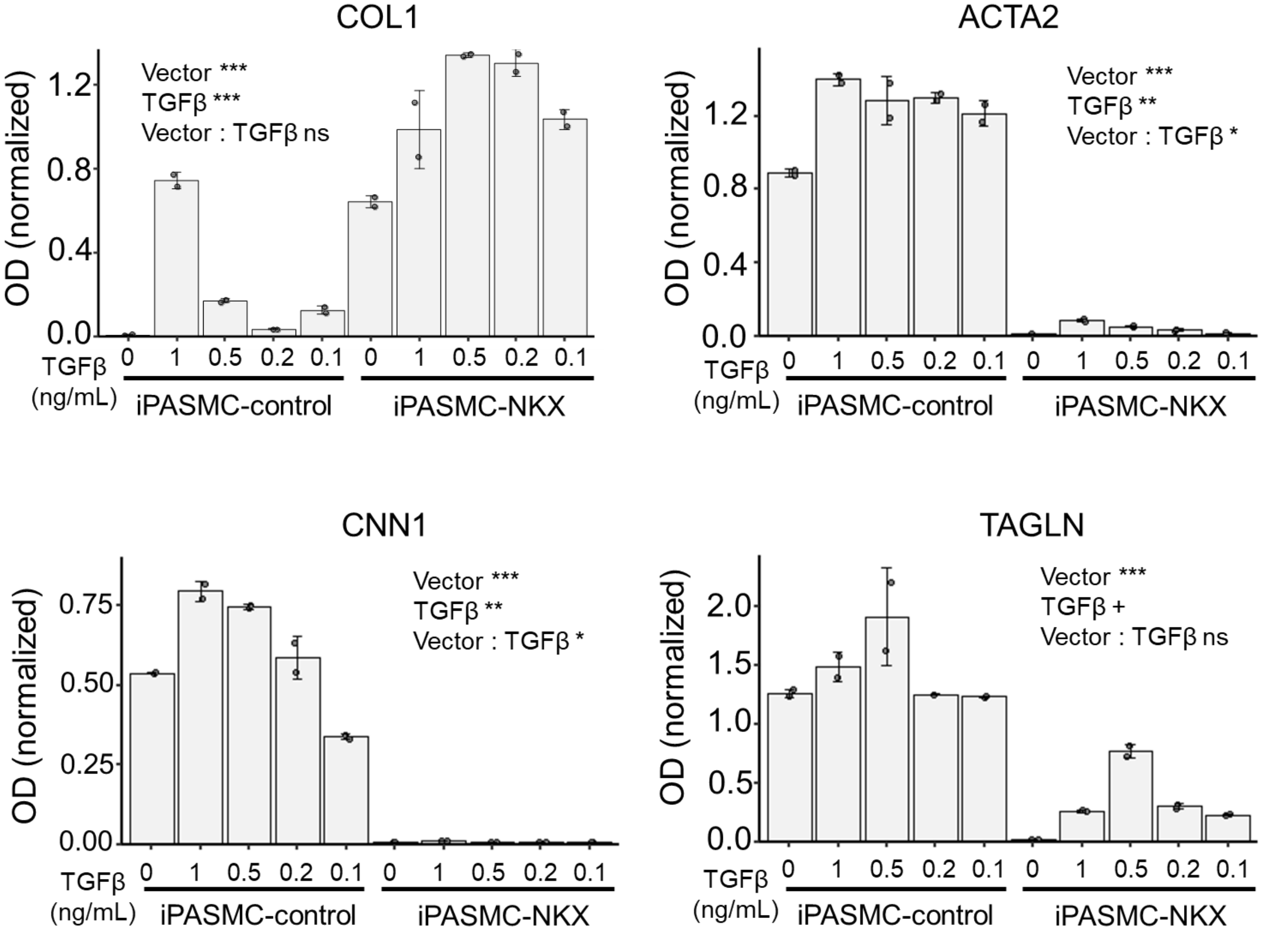

Supplementary Figure 10. Densitometry analysis for figure 4. Two independently obtained western blots from the same samples were analysed by densitometry. Optical density for COL1, ACTA2, CNN1 and TAGLN, was normalized to GAPDH (normalized OD). Statistical analysis was carried out by 2-way ANOVA using the expression vector used and the amount of TGFβ as the dependent variables. The control and NKX2-5 lentivirally transduced immortalized hPASMC are denoted by iPASMC-control and iPASMC-NKX respectively. The statistical significance of the Vector used, the amount of TGFβ added and their interaction factor (Vector:TGFβ) are indicated with the appropriate label. *** = $p<0.001$, ** = $p < 0.01$, * = $p < 0.05$, + = $p<0.1$
