# Supplementary material for: NKX2-5 regulates vessel remodeling in scleroderma-associated pulmonary arterial hypertension
Source: JCI Insight. 2024 Apr 23;9(10):e164191. doi: 10.1172/jci.insight.164191 (PMC11141943; doi:10.1172/jci.insight.164191)
Supplement: Unedited blot and gel images [file jciinsight-9-164191-s137.pptx]

## Slide 1
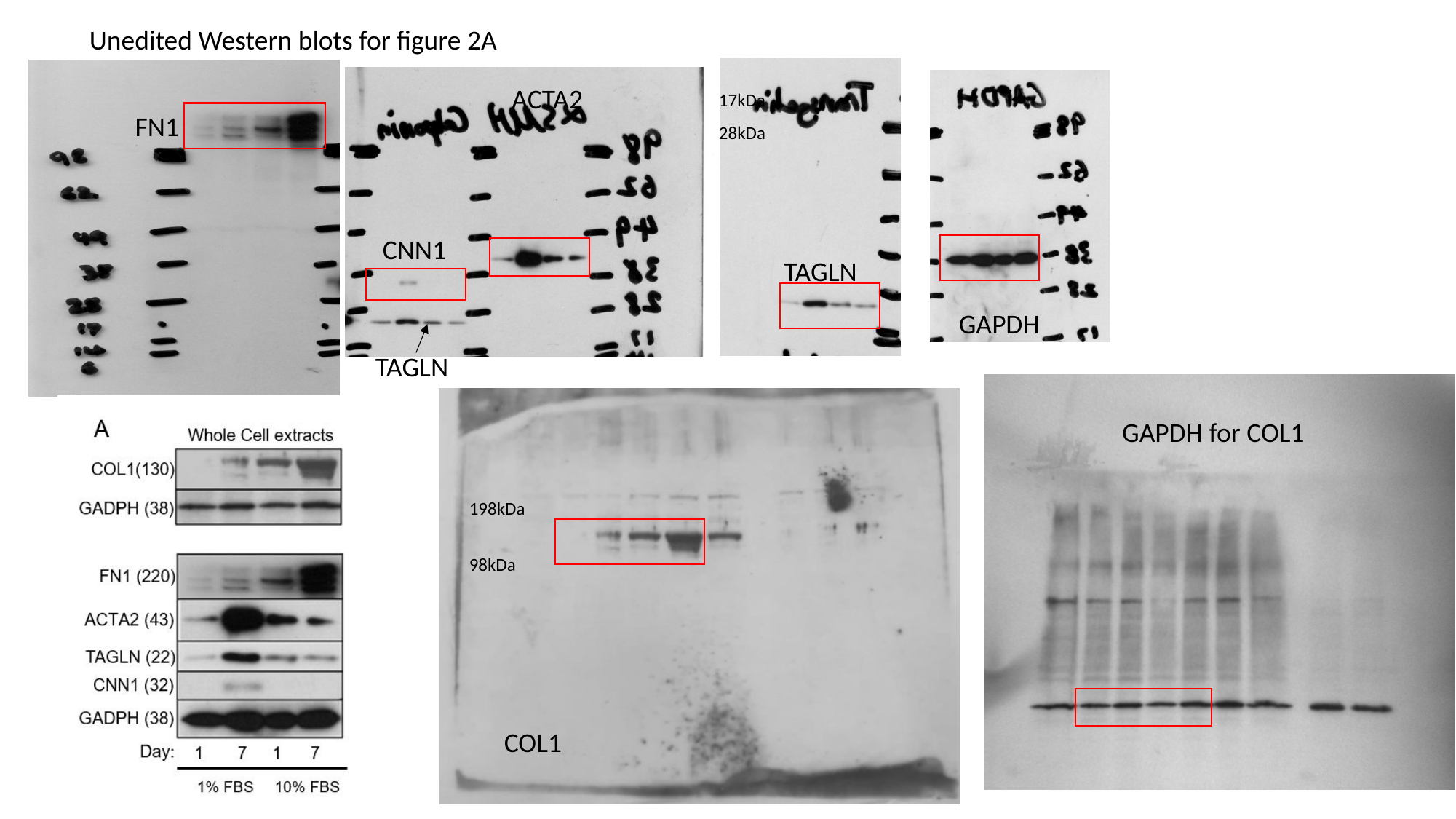

Unedited Western blots for figure 2A
ACTA2
17kDa
 FN1
28kDa
CNN1
TAGLN
GAPDH
TAGLN
GAPDH for COL1
198kDa
98kDa
COL1

## Slide 2
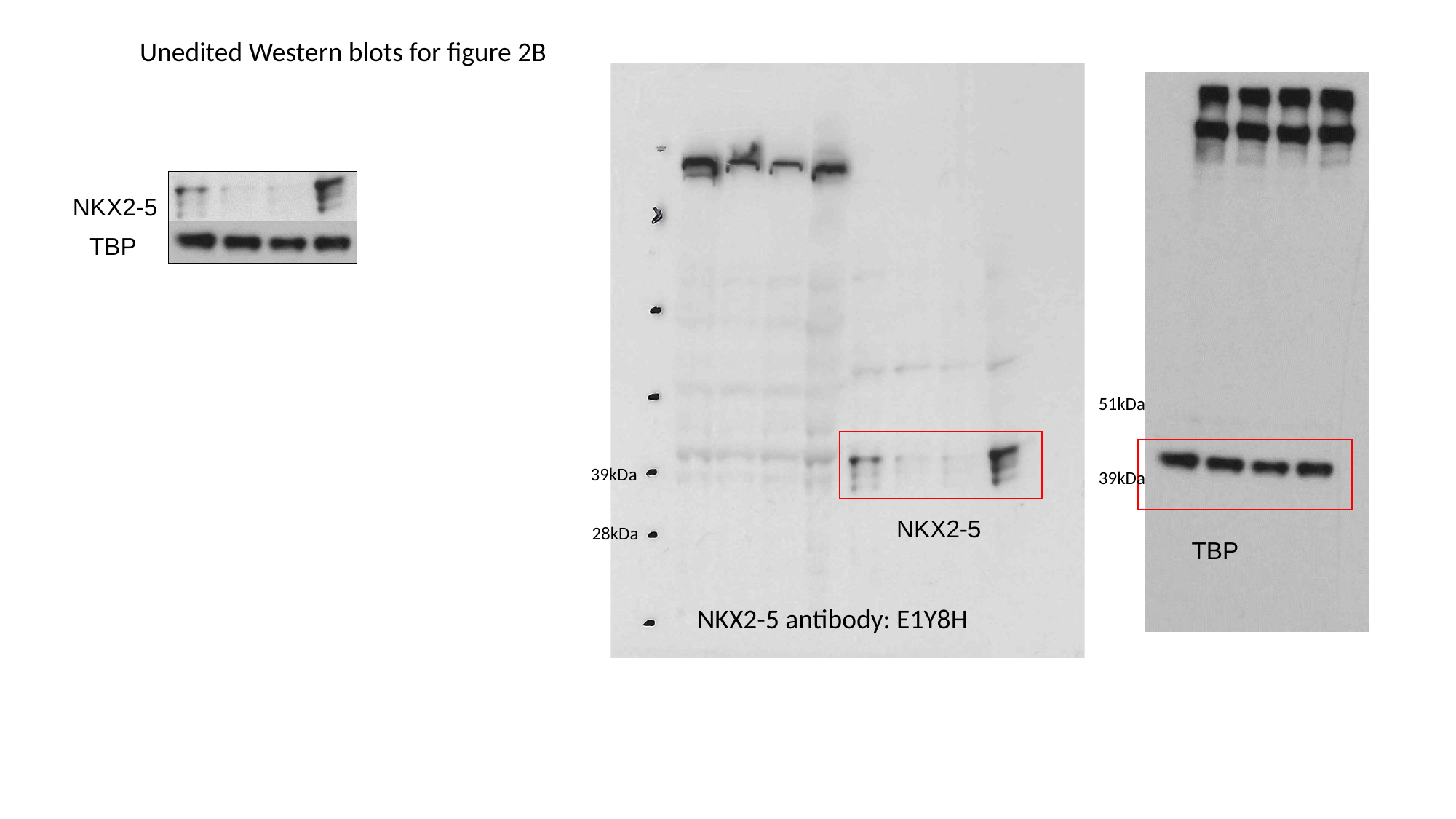

Unedited Western blots for figure 2B
NKX2-5
TBP
51kDa
39kDa
39kDa
NKX2-5
28kDa
TBP
NKX2-5 antibody: E1Y8H

## Slide 3
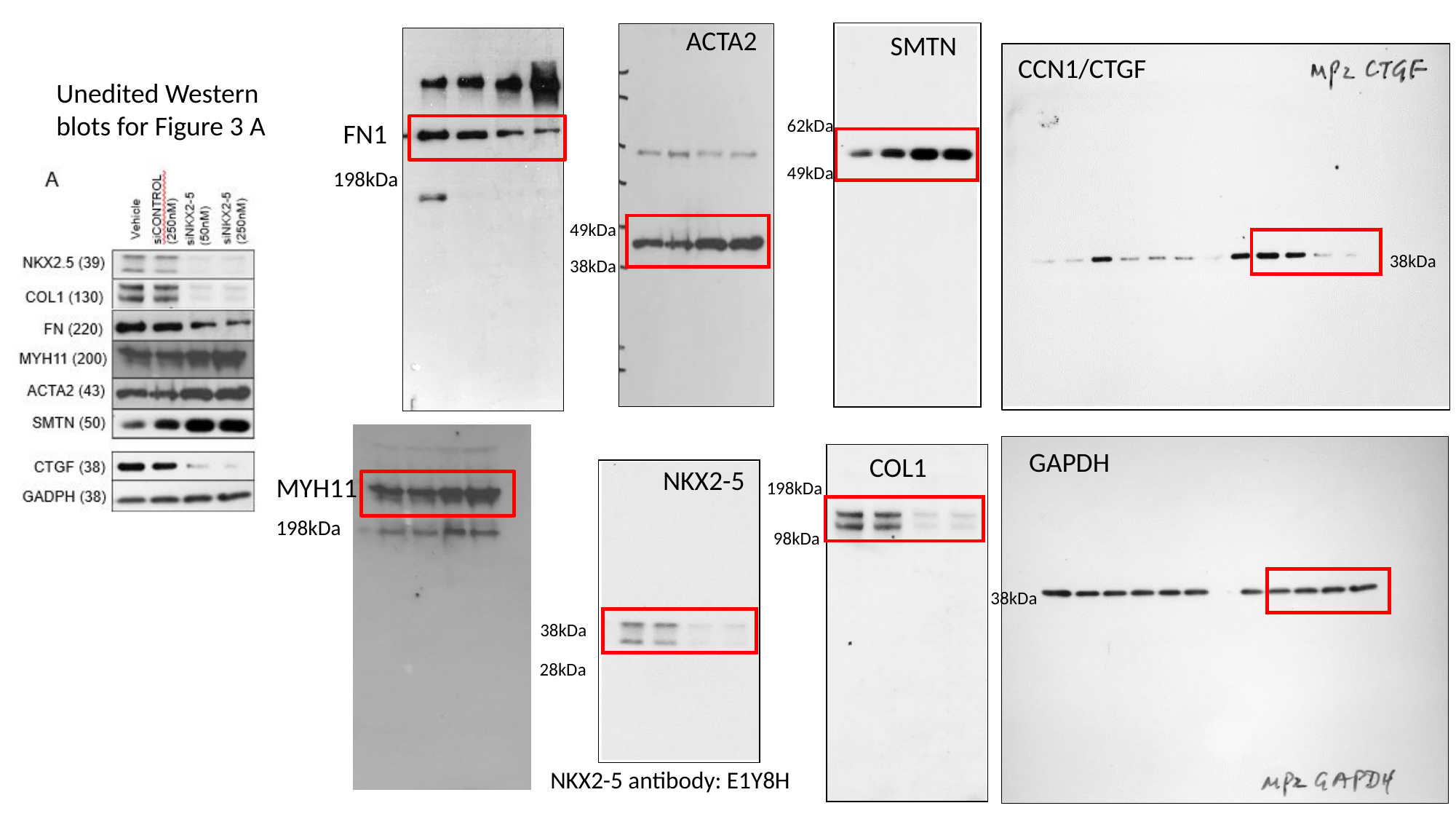

ACTA2
SMTN
CCN1/CTGF
Unedited Western blots for Figure 3 A
62kDa
FN1
49kDa
198kDa
49kDa
38kDa
38kDa
GAPDH
38kDa
COL1
198kDa
98kDa
NKX2-5
MYH11
198kDa
38kDa
28kDa
NKX2-5 antibody: E1Y8H

## Slide 4
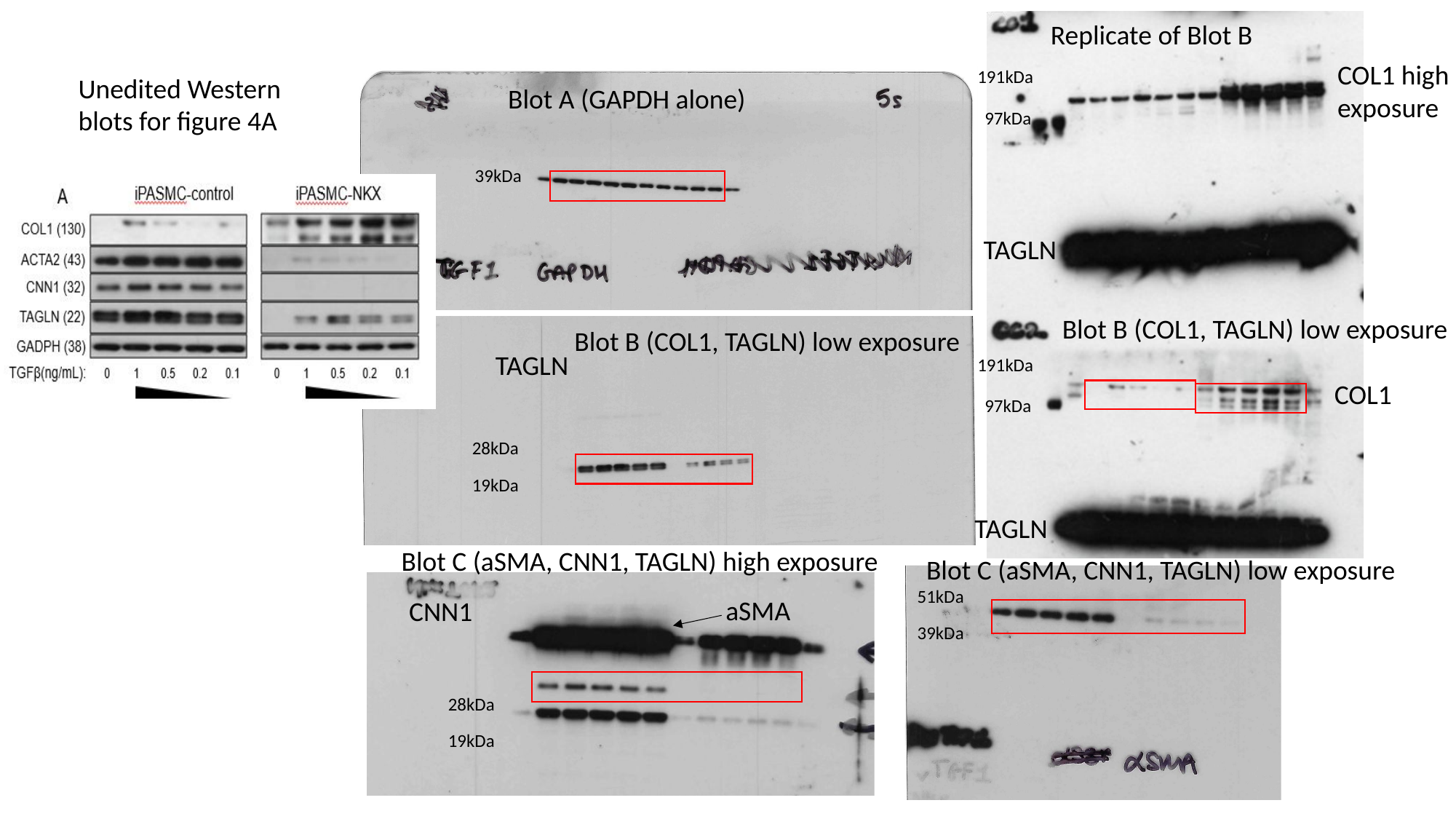

Replicate of Blot B
COL1 high exposure
191kDa
Unedited Western blots for figure 4A
Blot A (GAPDH alone)
97kDa
39kDa
TAGLN
Blot B (COL1, TAGLN) low exposure
Blot B (COL1, TAGLN) low exposure
TAGLN
191kDa
COL1
97kDa
28kDa
19kDa
TAGLN
Blot C (aSMA, CNN1, TAGLN) high exposure
Blot C (aSMA, CNN1, TAGLN) low exposure
51kDa
aSMA
CNN1
39kDa
28kDa
19kDa

## Slide 5
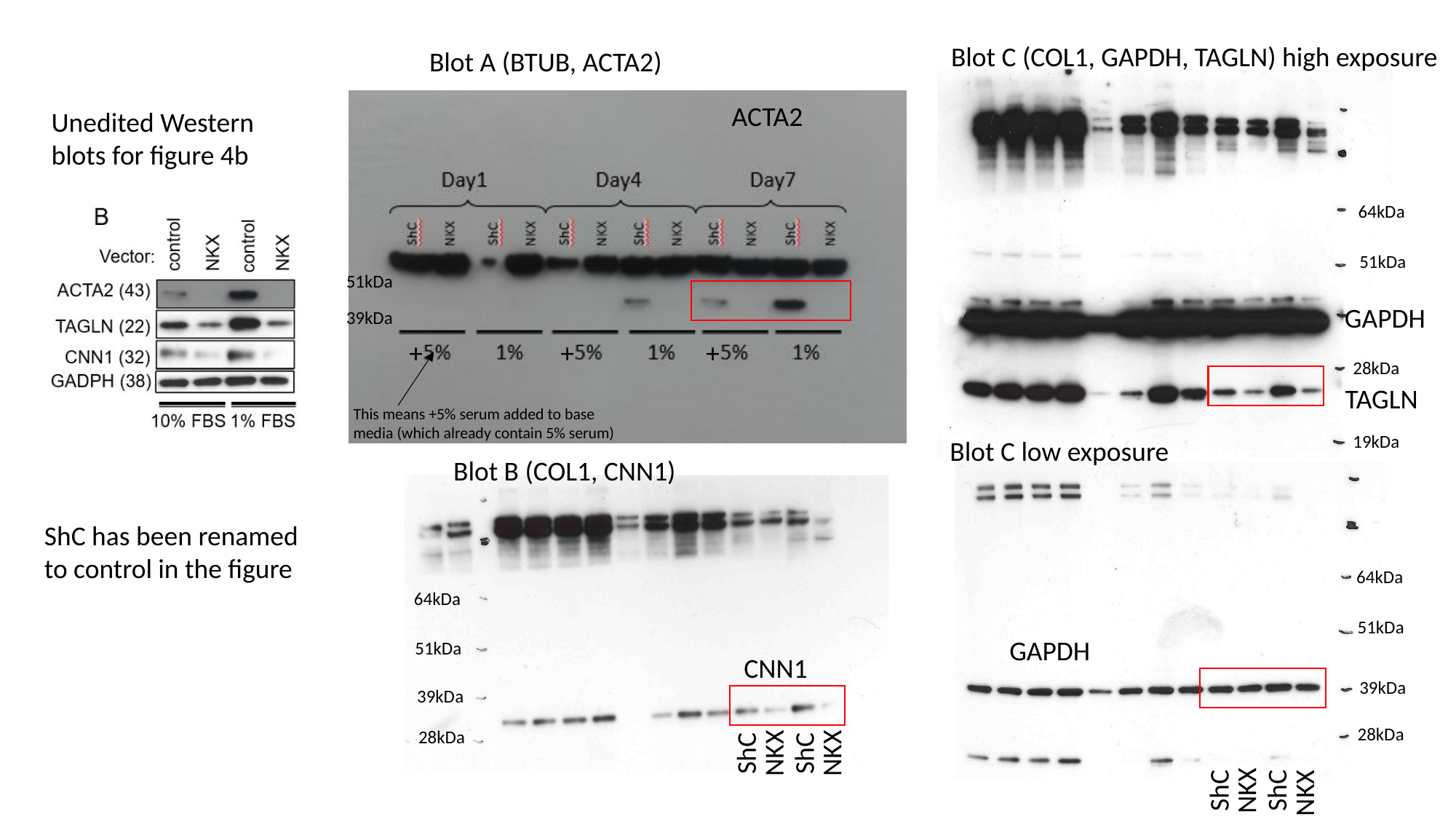

Blot C (COL1, GAPDH, TAGLN) high exposure
Blot A (BTUB, ACTA2)
ACTA2
Unedited Western blots for figure 4b
64kDa
51kDa
51kDa
GAPDH
39kDa
+
+
+
28kDa
TAGLN
This means +5% serum added to base media (which already contain 5% serum)
19kDa
Blot C low exposure
Blot B (COL1, CNN1)
ShC has been renamed to control in the figure
64kDa
64kDa
51kDa
GAPDH
51kDa
CNN1
39kDa
39kDa
28kDa
28kDa
ShC
NKX
ShC
NKX
ShC
NKX
ShC
NKX

## Slide 6
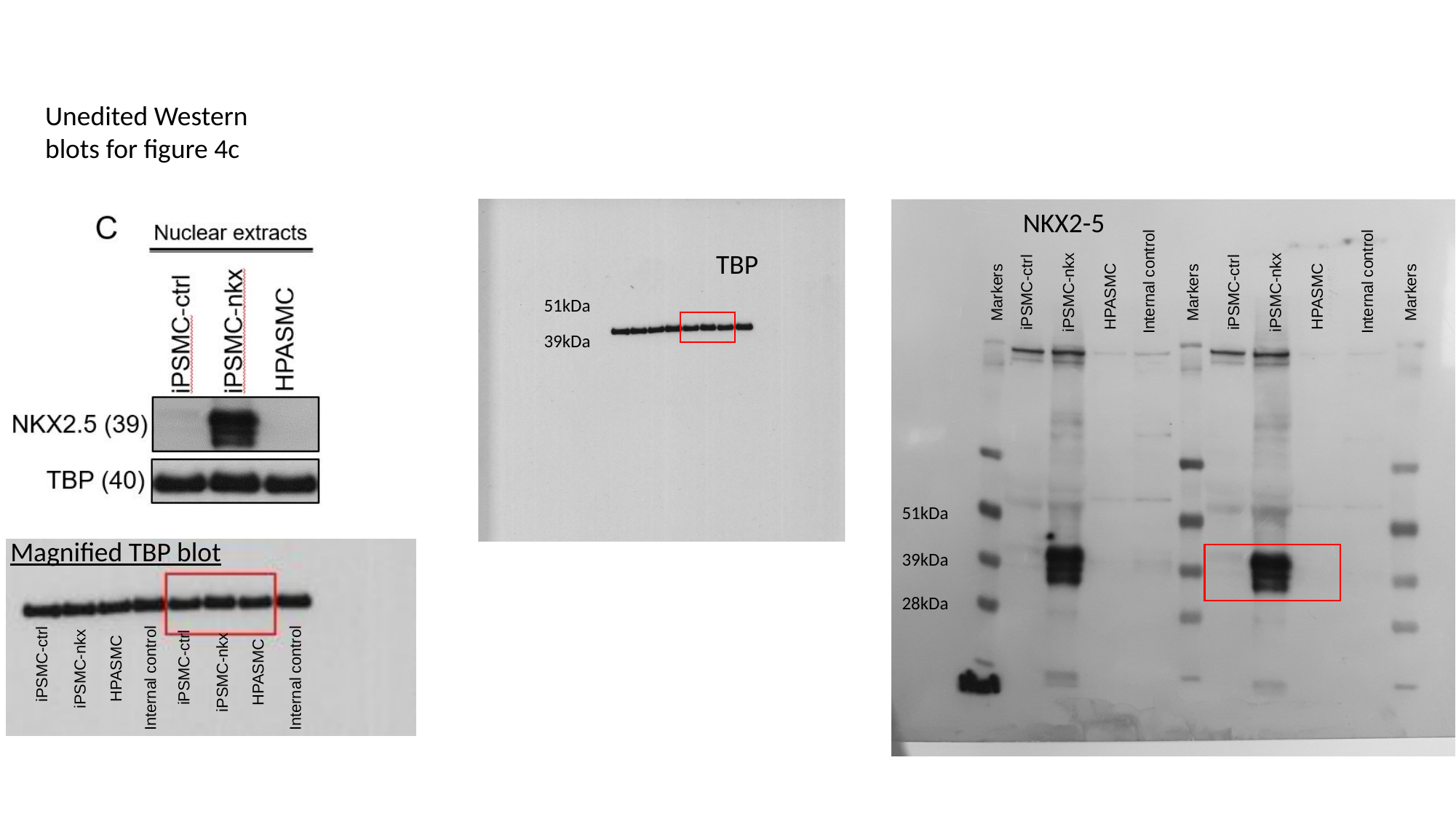

Unedited Western blots for figure 4c
NKX2-5
TBP
Internal control
Internal control
iPSMC-nkx
iPSMC-nkx
iPSMC-ctrl
iPSMC-ctrl
Markers
Markers
Markers
HPASMC
HPASMC
51kDa
39kDa
51kDa
Magnified TBP blot
iPSMC-ctrl
Internal control
Internal control
iPSMC-ctrl
HPASMC
iPSMC-nkx
HPASMC
iPSMC-nkx
39kDa
28kDa

## Slide 7
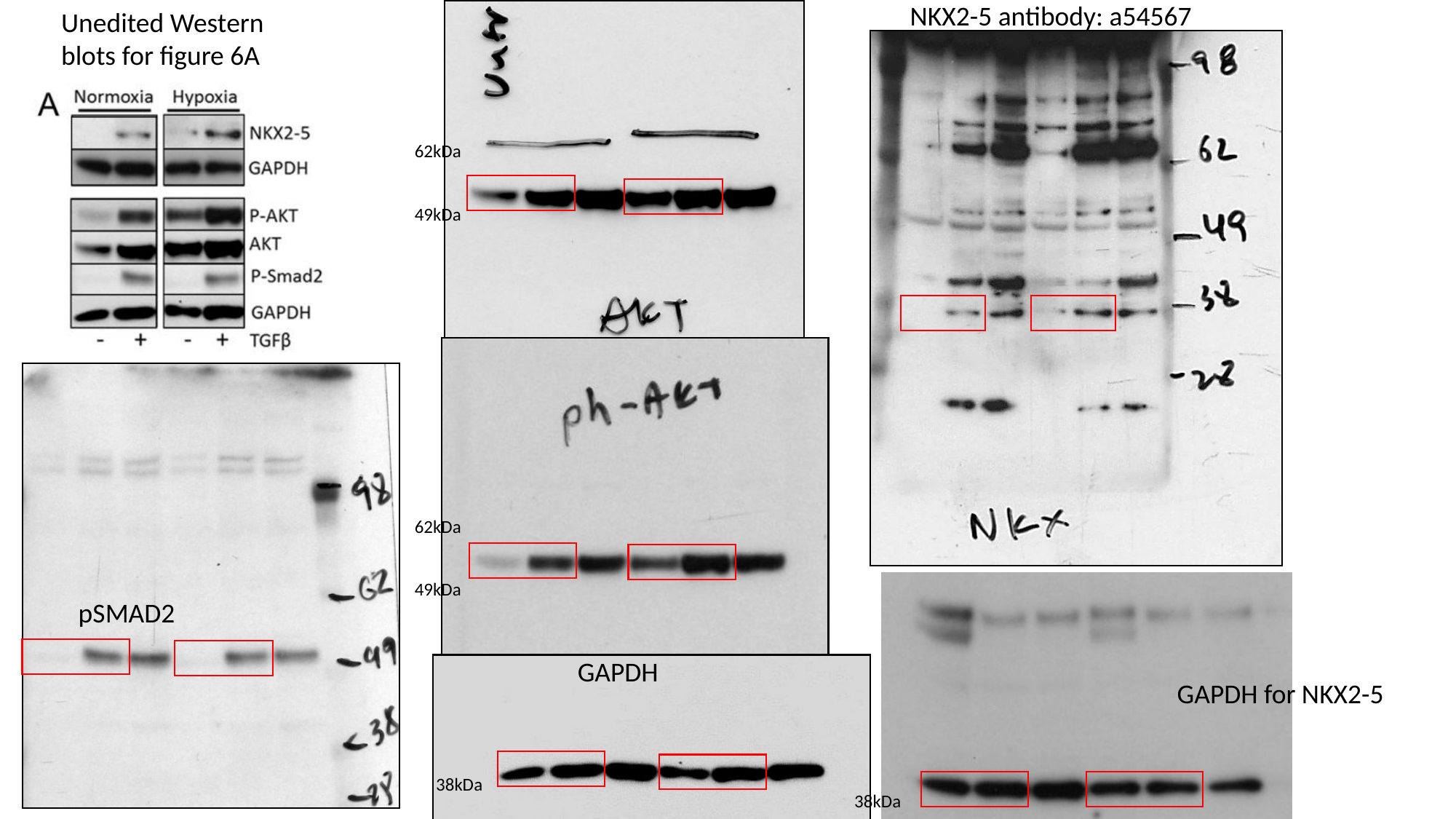

NKX2-5 antibody: a54567
Unedited Western blots for figure 6A
62kDa
49kDa
62kDa
49kDa
pSMAD2
GAPDH
GAPDH for NKX2-5
38kDa
38kDa

## Slide 8
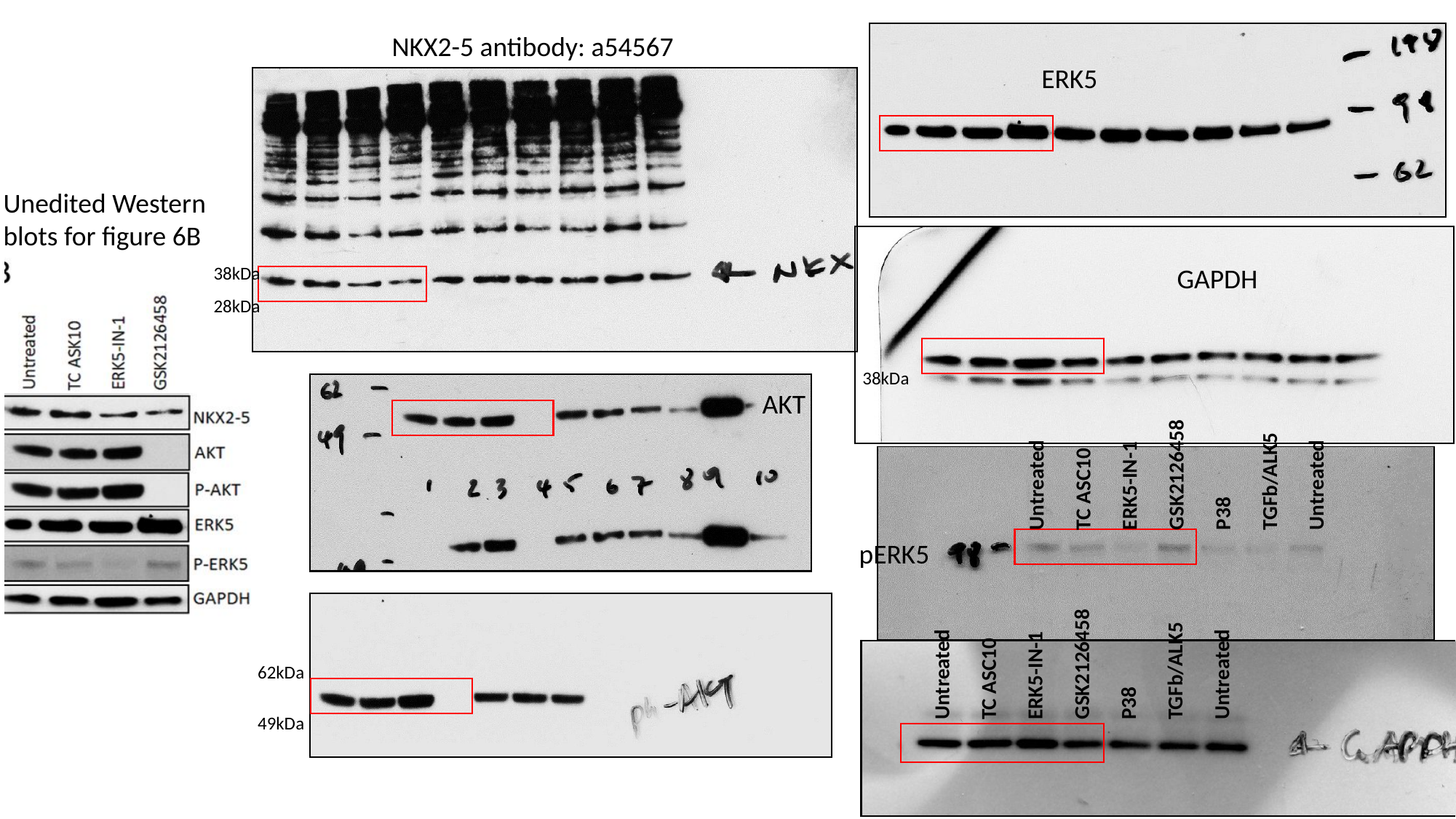

NKX2-5 antibody: a54567
ERK5
Unedited Western blots for figure 6B
GAPDH
38kDa
38kDa
28kDa
AKT
Untreated
TC ASC10
ERK5-IN-1
GSK2126458
P38
TGFb/ALK5
Untreated
pERK5
Untreated
TC ASC10
ERK5-IN-1
GSK2126458
P38
TGFb/ALK5
Untreated
62kDa
49kDa

## Slide 9
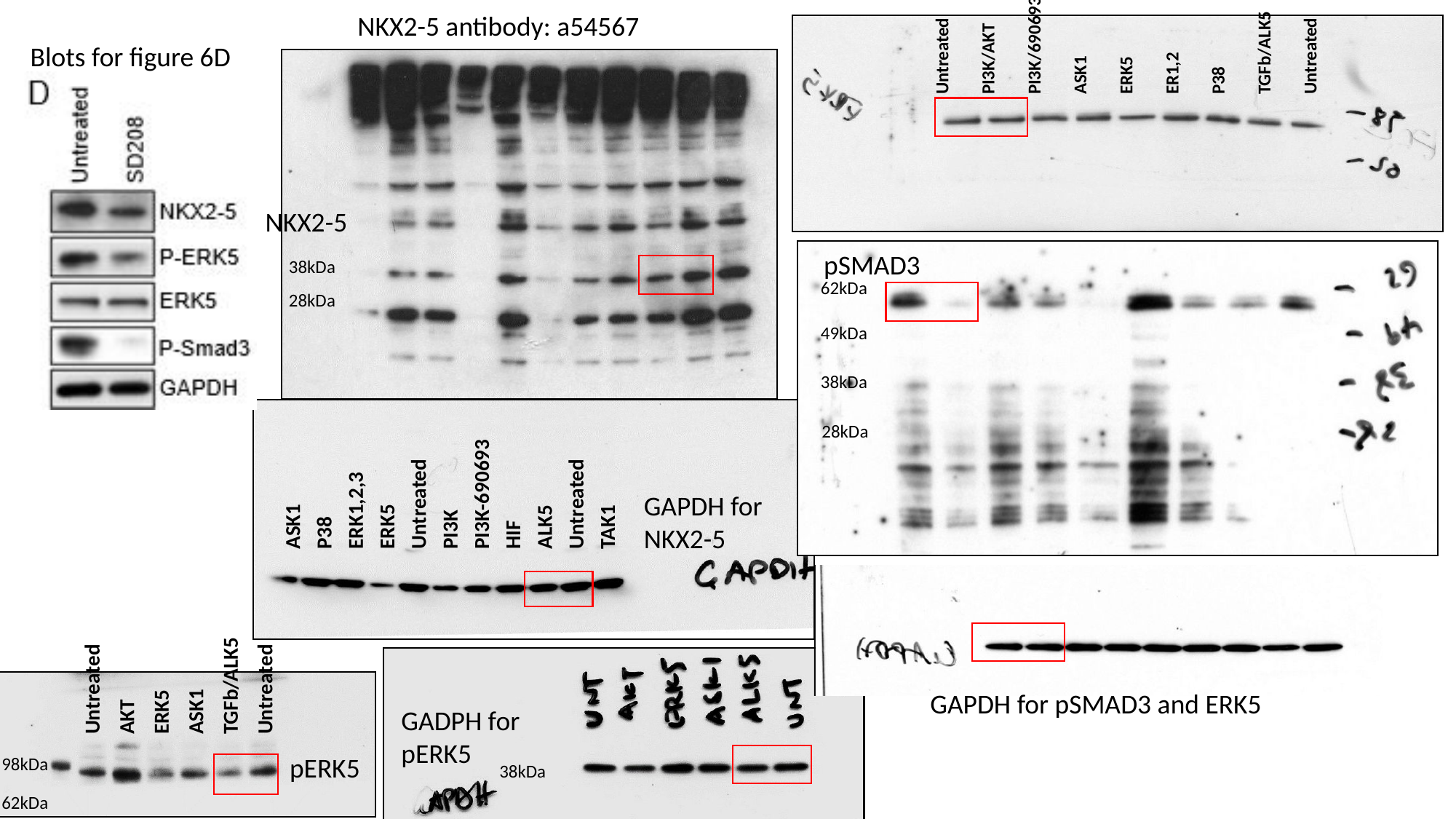

Untreated
PI3K/AKT
PI3K/690693
ASK1
ERK5
ER1,2
P38
TGFb/ALK5
Untreated
NKX2-5 antibody: a54567
Blots for figure 6D
NKX2-5
pSMAD3
62kDa
49kDa
38kDa
28kDa
38kDa
28kDa
ASK1
P38
ERK1,2,3
ERK5
Untreated
PI3K
PI3K-690693
HIF
ALK5
Untreated
TAK1
GAPDH for NKX2-5
Untreated
AKT
ERK5
ASK1
TGFb/ALK5
Untreated
GADPH for pERK5
38kDa
pERK5
98kDa
62kDa
GAPDH for pSMAD3 and ERK5

## Slide 10
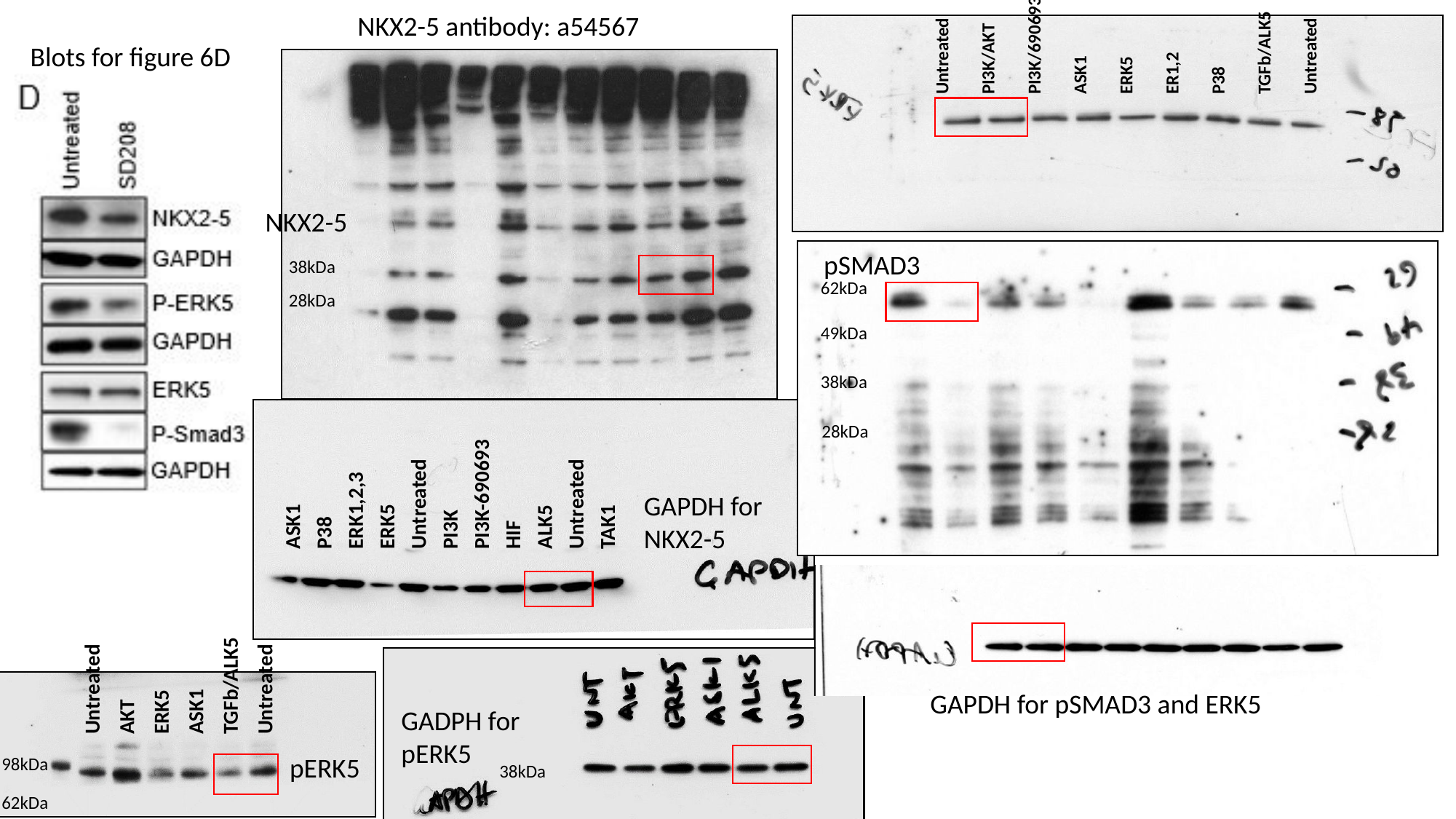

Untreated
PI3K/AKT
PI3K/690693
ASK1
ERK5
ER1,2
P38
TGFb/ALK5
Untreated
NKX2-5 antibody: a54567
Blots for figure 6D
NKX2-5
pSMAD3
62kDa
49kDa
38kDa
28kDa
38kDa
28kDa
ASK1
P38
ERK1,2,3
ERK5
Untreated
PI3K
PI3K-690693
HIF
ALK5
Untreated
TAK1
GAPDH for NKX2-5
Untreated
AKT
ERK5
ASK1
TGFb/ALK5
Untreated
GADPH for pERK5
38kDa
pERK5
98kDa
62kDa
GAPDH for pSMAD3 and ERK5

## Slide 11
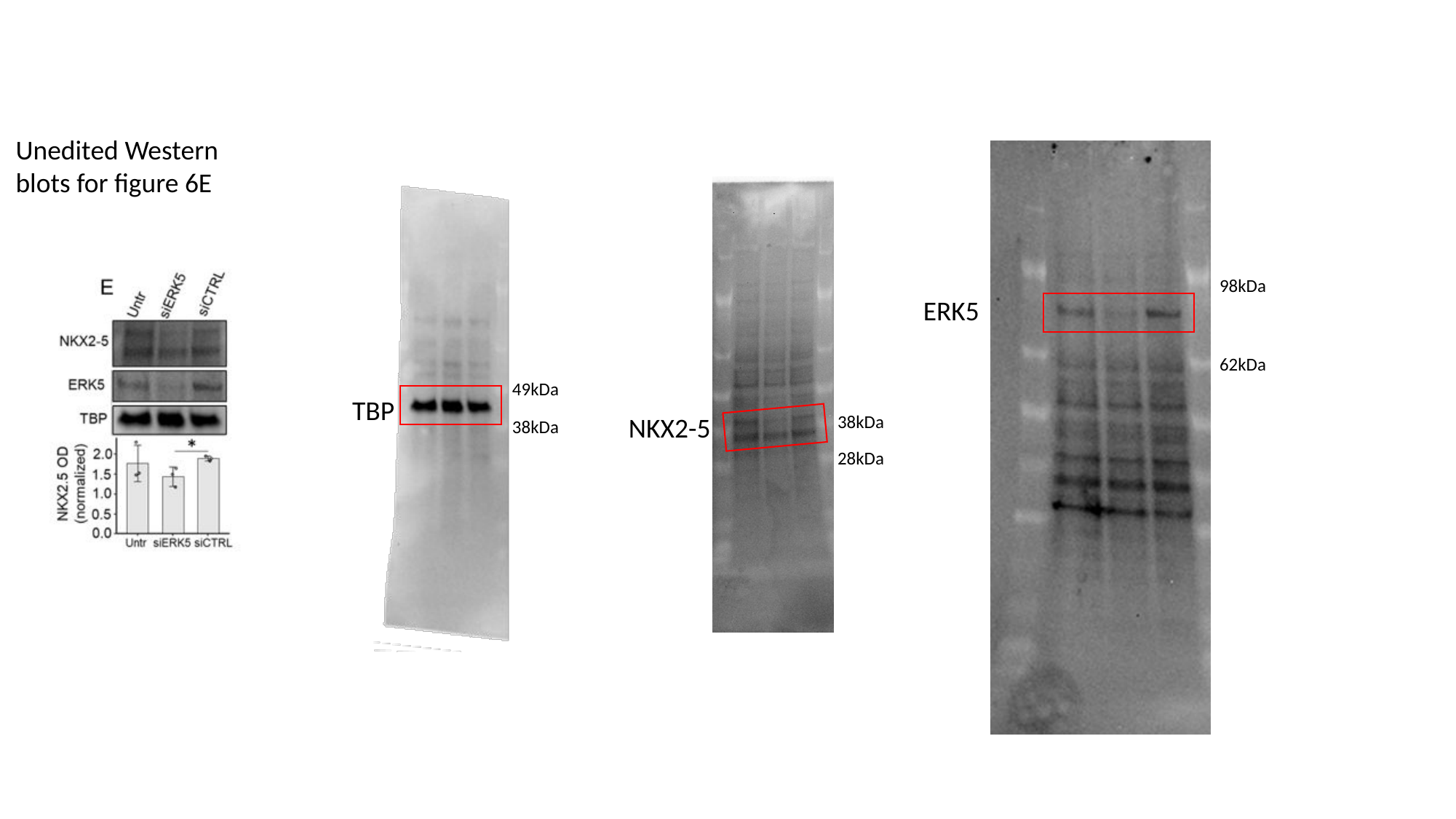

Unedited Western blots for figure 6E
98kDa
ERK5
62kDa
49kDa
TBP
38kDa
NKX2-5
38kDa
28kDa

## Slide 12
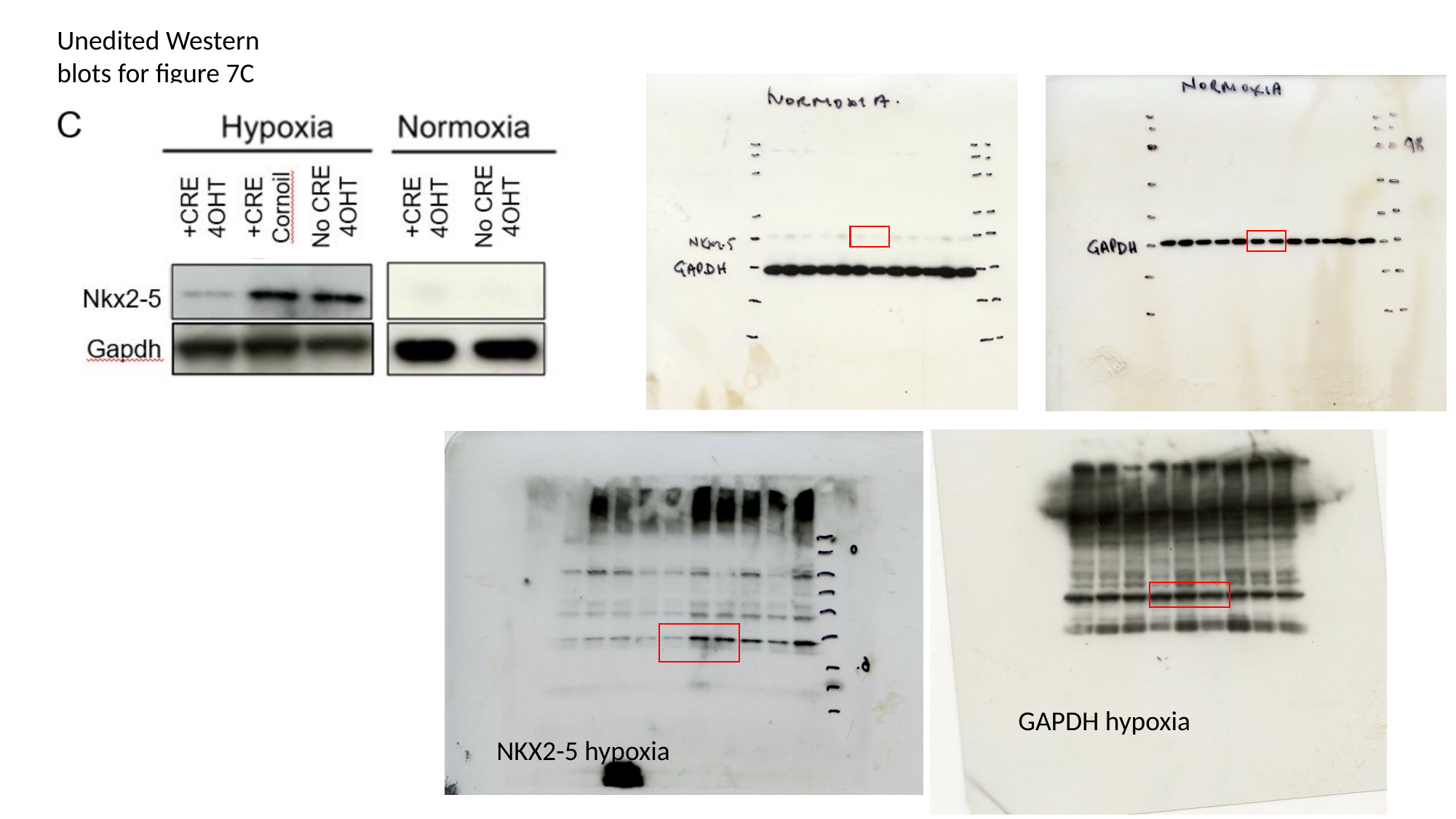

Unedited Western blots for figure 7C
GAPDH hypoxia
NKX2-5 hypoxia

## Slide 13
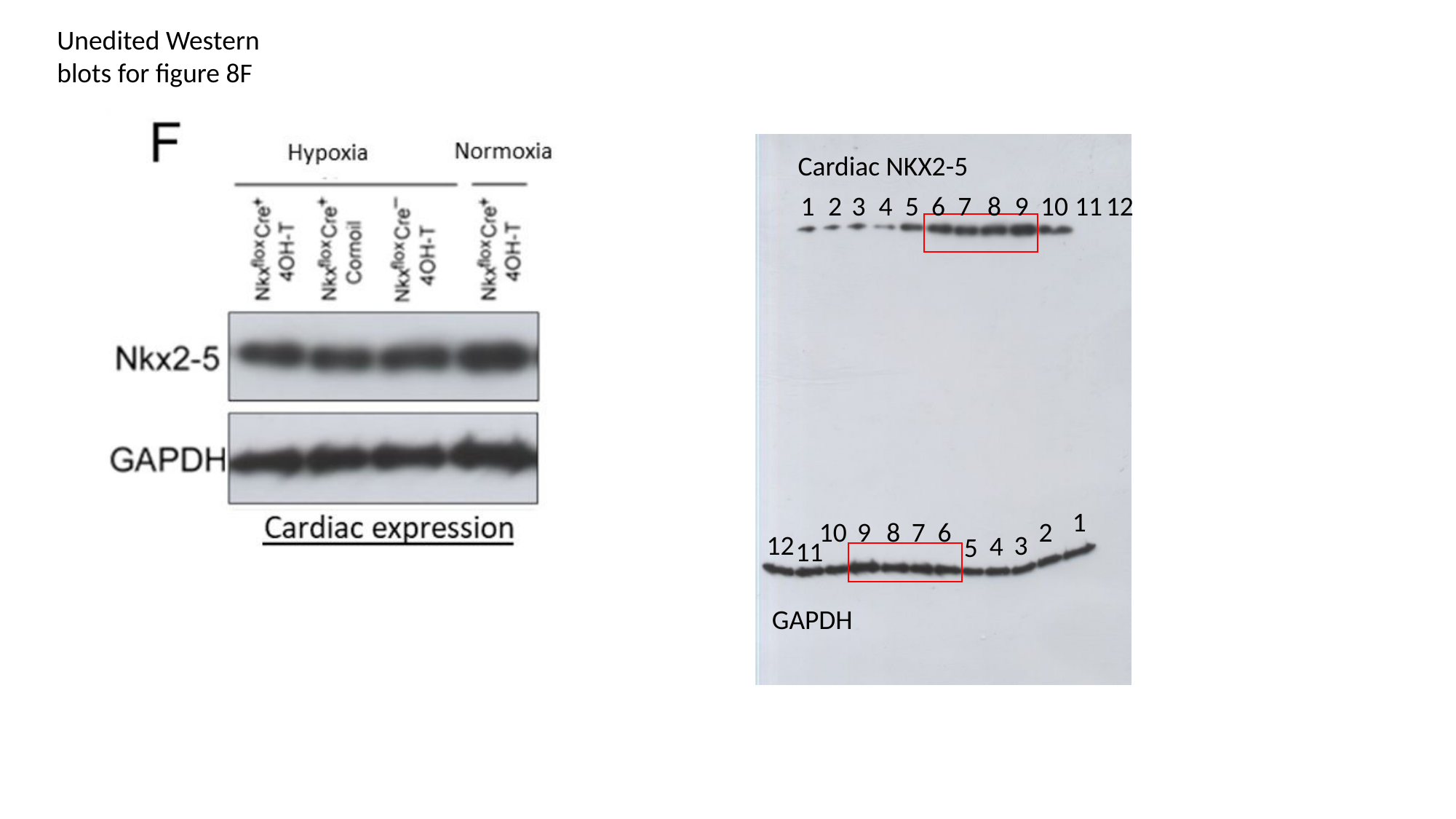

Unedited Western blots for figure 8F
Cardiac NKX2-5
1
2
3
4
5
6
7
8
9
10
11
12
1
10
9
8
7
6
2
12
3
4
5
11
GAPDH

## Slide 14
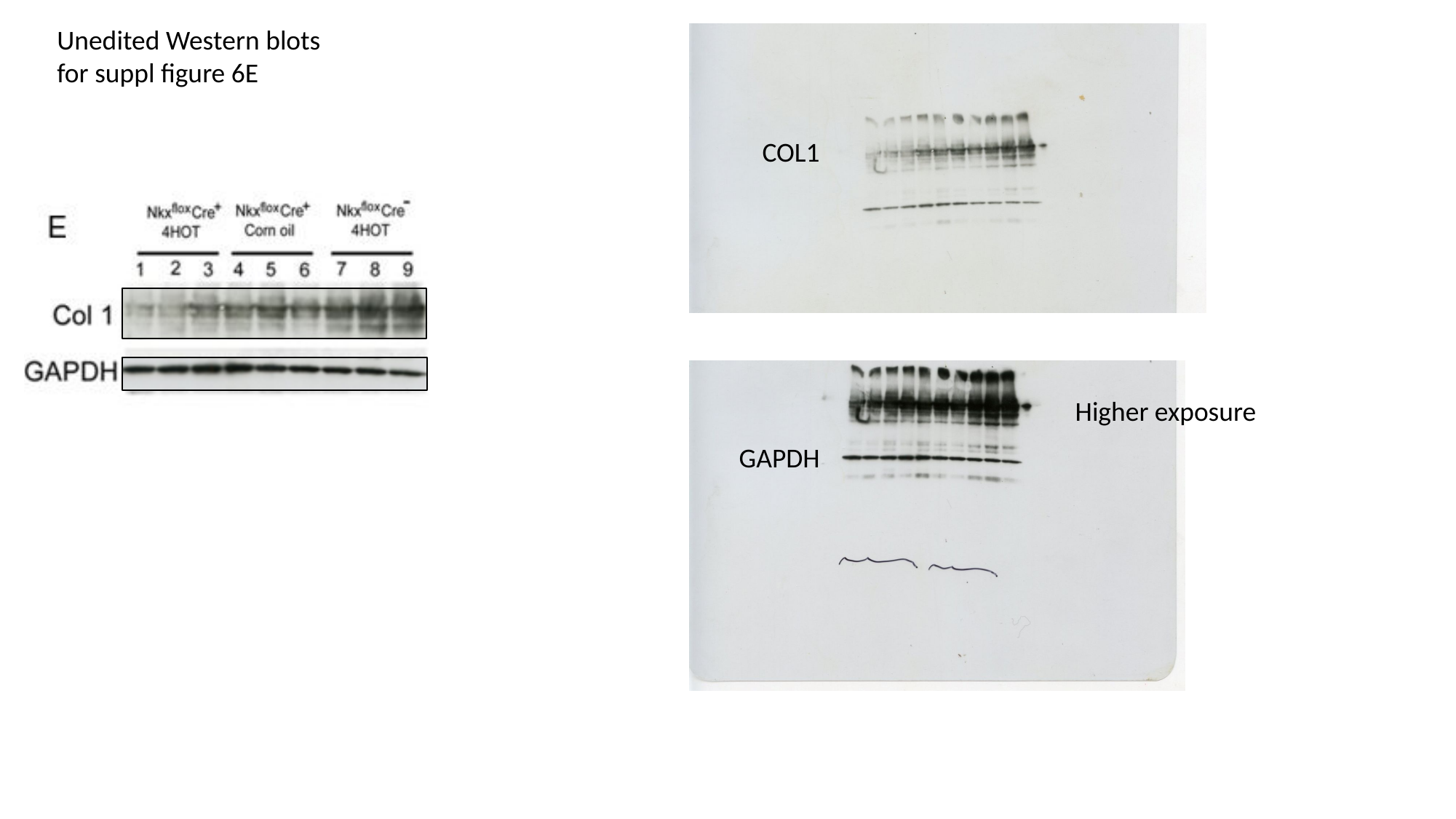

Unedited Western blots for suppl figure 6E
COL1
Higher exposure
GAPDH
